# Optimal progressivity of personal income tax: a general equilibrium evaluation for Spain

**DOI:** 10.1007/s13209-020-00226-0

**Published:** 2020-11-25

**Authors:** Darío Serrano-Puente

**Affiliations:** Banco de España, Madrid, Spain

**Keywords:** Income tax, Progressivity, Inequality, Income and wealth distribution, General equilibrium, Heterogeneous agents, D31, C68, E62, H21

## Abstract

Is the Spanish economy positioned at its optimal progressivity level in personal income tax? This article quantifies the aggregate, distributional, and welfare consequences of moving toward such an optimal level. A heterogeneous households general equilibrium model featuring both life cycle and dynastic elements is calibrated to replicate some characteristics of the Spanish economy and used to evaluate potential reforms of the tax system. The findings suggest that increasing progressivity would be optimal, even though it would involve an efficiency loss. The optimal reform of the tax schedule would reduce wealth and income inequality at the cost of negative effects on capital, labor, and output. Finally, these theoretical results are evaluated using tax microdata and describe a current scenario where the income-top households typically face suboptimal effective average tax rates.

## Introduction

Many modern governments implement a redistributive fiscal policy, where personal income is taxed at an increasingly higher rate, while transfers tend to target the poorest households. The taxation of personal income is not a minor issue, since most of the OECD economies obtain a large proportion of their tax collection through it.^1^ In Spain, there is an intense debate about how to finance the fiscal stimulus recovery plans to alleviate the economic consequences of the COVID-19 crisis and, more precisely, about how to deal with the unavoidable and needed fiscal consolidation process that will surely follow the enormous government fiscal effort. In particular, this growing political debate is taking up many headlines on the so-called “fiscal justice,” which is putting on the table a tax rate increase for the high-income earners, i.e., an increase in the progressivity of the personal income tax. These policies are initially developed to produce a more egalitarian distribution of income and, consequently, to provide social insurance for both currently living households that suffer from large income fluctuations, and for future generations that face uncertainty about what their initial state will be.

Raising taxes on higher incomes may be potentially justified by the increase in income and wealth inequality in recent years, especially after the 2007 crisis. These concerns over rising economic inequalities have resulted in a huge body of literature. One of the clearest examples is the paper by Piketty (2015), which triggered a widespread discussion on the nature and evolution of wealth inequalities worldwide. A recent study by Anghel et al. (2018) provides an overview of the inequality levels in Spain and their evolution. They show that the wave of unemployment caused by the 2007 crisis resulted in an inequality increase in per capita income. As for the wealth inequality in Spain, they show that it exceeds income inequality and it increased after the crisis, which may be due to financial assets outperforming real assets according to their vision. By international standards, Spain’s wealth inequality is moderate, as the ownership of real assets is more widespread than in other countries. Beforehand, one is likely to consider that raising taxes on the income-rich households could reverse the growing concentration at the top. However, this type of policy could be very costly in terms of efficiency in advanced market economies.

The optimal design of a redistributive tax system is subject to many constraints. Bakis et al. (2015) emphasize three of them in their study about the transitional dynamics of setting an optimal progressivity level. First, agents may have access to self-insurance through savings or bequests, and then, increasing the redistributiveness of the fiscal policies would alleviate the need for such self-insurance and would crowd out capital accumulation, leading to reduced investment and output. Second, misinformation may prevent government from observing individual productivity and, by raising taxes on certain agents, it could provoke incentive problems that discourage labor and thereby reduce output. Third, large-scale shifts in labor and capital supply (savings) alter the wage rate and the interest rate, which may have unexpected repercussions for income redistribution.

This is why having a quantitative theory that accounts accurately for the observed income and wealth inequality is crucial when assessing the aggregate, distributional, and welfare implications of certain policies. For that purpose, a heterogeneous households general equilibrium model is here calibrated to replicate some characteristics of the Spanish economy and used to compare the steady-state consequences of setting an optimal progressivity level in the Spanish personal income tax. For the Spanish case, general equilibrium models with heterogeneous agents have already been used to examine the effects of certain reforms.^2^ However, not many studies are encountered to use general equilibrium models with heterogeneous agents to explore the relationship between fiscal policy variables and the endogenous cross-sectional distribution of income and wealth in Spain, in turn the main topic of analysis of this study. The references that we find with respect to this concern are mentioned in the following lines. Pijoan-Mas and González Torrabadella (2006) quantify the aggregate and distributional implications of an array of revenue neutral flat tax reforms for Spain. Viegas and Ribeiro (2015) attempt to characterize the Spanish debt consolidation process in order to assess its effects on economic inequality and welfare. And finally, Guner et al. (2020) uses a life cycle model to evaluate the impact on fiscal revenues of changes in the progressivity of personal income taxes in Spain. The study herein presented is closely related to that research, but the focus in the present work is on the welfare implications of the reforms.

In general, the literature on optimal taxation in a general equilibrium framework is vast, but these pieces of work do not have their focus on the Spanish context. Kindermann and Krueger (2018), Conesa et al. (2009), and Guner et al. (2017) study the effects of taxing higher incomes and particularly assess whether and to what extent capital should be taxed. Moreover, Bakis et al. (2015), Heathcote et al. (2017), and Conesa and Krueger (2006) provide an assessment of the optimal progressivity of personal income tax and how redistributive the government’s fiscal policy should be. Finally, Díaz-Giménez and Pijoan-Mas (2019) evaluate the gains that a progressive consumption tax could have with the same modeling framework that is used in the present analysis. Hence, due to the topic and the underlying methodology, the work contained herein would contribute to this body of literature.

Again, although some previous studies have analyzed the Spanish economy with general equilibrium models devoted to study policy implications for wealth and income inequality, none of them combined the main characteristics of the dynastic and of the life cycle abstractions (hybrid model with retirement and bequests). Contrarily, these models are built in either dynastic or life cycle fashions. This is where this study adds value, as it proposes other methodologies previously used in other contexts to be applied to the Spanish scenario. The theoretical framework is built for Spain following Castañeda et al. (2003), who also rely on a hybrid approach to account for the US earnings and wealth inequality.^3^ Heterogeneity is introduced in this setup via distinct labor market opportunities using an uninsurable process on the endowment of efficiency labor units that features nonlinear dynamics. Given the labor market opportunity, the households choose their work effort. In other words, the labor choice is set here to be endogenous, as in Pijoan-Mas and González Torrabadella (2006). Life cycle characteristics are modeled using aging and retirement, and dynastic links are modeled in a way that households are altruistic toward their descendants. Once the model is properly calibrated to match some empirical statistics of the Spanish economy, these features ensure that households save for precautionary motives (life cycle reasons and altruistic reasons), as argued by Díaz-Giménez and Pijoan-Mas (2019). This model economy replicates the distributions of income and wealth in very much detail. Further, it also works well when replicating the very top tails of those distributions.^4^

Further, once the theoretical framework is defined, a bunch of potential progressivity reforms are assessed through the calculation of many different general equilibria (one equilibrium for each degree of progressivity evaluated). Then a Benthamite social planner, who takes into account all households in the economy by putting the same weight on each of them, discerns the optimal progressivity reform. The findings suggest that aggregate social welfare is maximized when the level of progressivity of the Spanish personal income tax is increased to some extent. More precisely, in the optimally reformed scenario (setting the optimal level of progressivity), welfare gains are equivalent to an average increase of 3.08% of consumption.

By decomposing the aggregate welfare change, it is shown that most of the welfare gains are obtained by direct improvements in the tax system. It means that most of the aggregate welfare gains come from a majority of households facing a lower tax rate, i.e., the poorest households facing lower effective income tax rates and richest households affronting higher effective income tax rates. On the contrary, the general equilibrium effects of the optimal reformed economy (higher interest rate and lower wage) and the effects resulting from changes in the equilibrium distribution of households across income levels (larger mass of households at lower income levels) show a welfare loss, but these losses are so small that together cannot overpass the welfare gains directly coming from the reformed tax system, jointly resulting in positive aggregate welfare changes. These welfare gains are decomposed by household type, where it is observed that the poorest working and non-working households are the ones who benefit the most from the reform. Contrarily, the most efficient working households and the wealthiest ones (either working or non-working) are those who experience the largest trade-off between (i) positive welfare effects derived from higher income (due to an increased interest rate that pushes up capital returns) and (ii) adverse effects emerging from higher tax payments (due to the increase in progressivity of the income tax that discourages labor and savings). The losses from this trade-off are particularly high in top parts of the income and wealth distributions and clearly offset the potential welfare gains of the households populating such areas. Therefore, knowing that these agents would be the losers of the reform, despite positive aggregate welfare effects, the consequences on aggregate capital, labor, and output would be negative, which means that the economy would experience an efficiency loss. Moreover, looking at the distributional implications, this reform would reduce income and wealth inequality.

Finally, the theoretical results are evaluated with Spanish tax microdata. From the point of view of a Benthamite social planner, households between the 20th and the 80th percentiles would experience a decrease in their average tax rates under the optimal progressivity reform. For example, the effective average tax rate encountered by a household situated within the 40th and the 60th percentiles of the income distribution would drop from 0.067 to 0.056, which involves a change of 1.1 p.p.. On the other hand, households above the 80th percentile would experience a drastic increment in their effective average tax rate. For instance, the top 1% households of the gross income distribution would go from confronting an average tax rate of 0.284 in the actual scenario to dealing with an average tax rate of 0.330 in the optimal one.

The remainder of the paper is structured as follows. The model is formally introduced in Sect. 2. Section 3 describes how the model has been calibrated to match Spanish aggregate and distributional data. Section 4 presents the optimal reform of the progressivity based on a welfare comparison between steady-states and details aggregate and distributional implications. Finally, Sect. 5 concludes.

## The model economy

The model economy analyzed in this study strictly follows the setup proposed by Castañeda et al. (2003), which is a modified version of the stochastic neoclassical growth model with uninsured idiosyncratic risk and no aggregate uncertainty. The main features of this theoretical framework can be summarized in as follows: (i) there is a number of households that are ex-ante identical (they all exhibit the same preferences); (ii) these households are differentiated among themselves by the uninsured household-specific shock that they receive in their endowments of efficiency labor units; (iii) households go through the life cycle and can be either workers or retirees (which can be interpreted as households out of the labor market in general); (iv) once households are retired, they face a probability of dying, and if they die, they are replaced by working-age descendants; and (v) households are altruistic toward their descendants.

### Population and endowment dynamics

This particular model economy is inhabited by a measured one continuum of heterogeneous dynastic households. The households can be either of working-age or retired, and they are all endowed with $\ell$ units of disposable time each period. Workers face an uninsured idiosyncratic stochastic process that determines their endowment of efficiency labor units. They also face an exogenous positive probability of retiring. Retired households are endowed with zero efficiency labor units and face an exogenous positive probability of dying. When a retired household dies, it is replaced by a working-age descendant that inherits the deceased household estate and, possibly, some of its earnings abilities. To denote the household’s random age and random endowment efficiency labor units jointly, a one-dimensional shock, *s*, is used. This process is assumed to be *i*.*i*.*d*. across households and follows a finite state Markov chain with conditional transition probabilities given by $\Gamma _{{\mathcal {S}}\mathcal {S'}}=\Gamma \left( s'|s\right) =Pr\left\{ s_{t+1}=s'|s_{t}=s\right\}$, where *s* and $s'\in {\mathcal {S}}=\{1,2,\ldots ,n\}$.

It is assumed that *s* takes values in one of two possible *J*-dimensional sets, $s\in {\mathcal {S}}={\mathcal {E}}\cup {\mathcal {R}}={1,2,\ldots ,J}\cup {J+1,J+2,\ldots ,2J}$. When a household draws shock $s\in {\mathcal {E}}$, it is of working-age and endowed with $e(s)>0$ efficiency labor units. When a household draws shock $s\in {\mathcal {R}}$, it is retired and endowed with zero efficiency labor units. When a household’s shock changes from $s\in {\mathcal {E}}$ to $s' \in {\mathcal {R}}$, the household has retired. When it changes from $s\in {\mathcal {R}}$ to $s'\in {\mathcal {E}}$, the retired household dies and is replaced by a working-age descendant that inherits the estate, $a_{t}$, that the deceased household had at the end of period *t*. Therefore, the joint age and endowment process imply that the transition probability matrix $\Gamma _{{\mathcal {S}}\mathcal {S'}}$ controls (i) the demographics of the model economy by determining the expected durations of the households’ working lives and retirements, (ii) the lifetime persistence of earnings by determining the mobility of households between states in ${\mathcal {E}}$, (iii) the life cycle pattern of earnings by determining how the endowments of efficiency labor units of new entrants differ from those of senior workers, and (iv) the intergenerational persistence of earnings by determining the correlation between the states in ${\mathcal {E}}$ for consecutive members of the same dynasty.

Since it is assumed that the presented joint age and endowment process takes values in two *J*-dimensional sets, the number of realizations of such process is 2*J*. Therefore, to specify the process on *s*, the values of $(2J)^2+J$ parameters must be chosen. $(2J)^2$ of these parameters are the conditional transition probabilities and the remaining *J* are the values of the endowment of efficiency labor units. However, some assumptions about the nature of the joint age and endowment process impose some additional structure/restrictions on the transition probability matrix, $\Gamma _{{\mathcal {S}}\mathcal {S'}}$, which reduce the large number of parameters. In order to understand these restrictions better, it helps to consider the following partition of this matrix:

1 $$\begin{aligned} \Gamma _{{\mathcal {S}}\mathcal {S'}}=\left[ \begin{matrix}\Gamma _{{\mathcal {E}}{\mathcal {E}}}&{}\quad \Gamma _{{\mathcal {E}}{\mathcal {R}}}\\ \Gamma _{{\mathcal {R}}{\mathcal {E}}}&{}\quad \Gamma _{{\mathcal {R}}{\mathcal {R}}}\end{matrix}\right] \end{aligned}$$

Submatrix $\Gamma _{{\mathcal {E}}{\mathcal {E}}}$ describes the changes in the endowments of efficiency labor units of working-age households that are still of working age one period later, and no restrictions are placed on it so the values of $J^2$ parameters must be chosen. Submatrix $\Gamma _{{\mathcal {E}}{\mathcal {R}}}$ describes the transitions from the working-age states into the retirement states. This submatrix is defined by $\Gamma _{\mathcal {ER}}:=p_{r} {\mathbf {I}}$, where $p_{r}$ is the probability of retiring and ${\mathbf {I}}$ is the identity matrix. This is because every working-age household faces the same probability of retiring and because only the last realization of the working-age shock is used to keep track of the earnings ability of the retirees. Consequently, the value of only one parameter must be chosen. Submatrix $\Gamma _{\mathcal {RR}}$ describes the changes in the retirement states of retired households that are still retired one period later. This submatrix is defined by $\Gamma _{{\mathcal {R}}{\mathcal {R}}}:=p_{s} {\mathbf {I}}$, where $(1-p_{s})$ is the probability of dying or exiting the economy. This is because the type of retired households never changes, because every retiree faces the same probability of dying or exiting the economy. Therefore, the value of only one parameter is needed to identify this submatrix. Finally, submatrix $\Gamma _{{\mathcal {R}}{\mathcal {E}}}$ describes the transitions from the retirement states into the working-age states that take place when a retired household dies and is replaced by its working-age descendant. The rows of this submatrix contain a two-parameter transformation of the stationary distribution of $s\in {\mathcal {E}}$, which is denoted by $\gamma ^{*}_{{\mathcal {E}}}$. Intuitively, the transformation amounts to shifting the probability mass from $\gamma ^{*}_{{\mathcal {E}}}$ toward both the first row of $\Gamma _{{\mathcal {R}}{\mathcal {E}}}$ and toward its diagonal. This particular transformation is aimed to control for both the life cycle profile of earnings and its intergenerational correlation. In short, to characterize $\Gamma _{{\mathcal {R}}{\mathcal {E}}}$, one must choose the value of the two shifting parameters, as it will explained in what follows.

*Intergenerational transmission of earnings ability* The process driving the transition between retirement and working-age states, i.e., transition submatrix $\Gamma _{{\mathcal {R}}{\mathcal {E}}}$, must reflects somehow the correlation between the average income of one generation and the average income of its immediate descendants. In this way, the working-age descendant that will replace a deceased retired household will inherit some earnings ability from its predecessor. It implies that the model economy here presented will capture the intergenerational persistence of earnings. Following the procedure presented in Castañeda et al. (2003), in order to determine such intergenerational persistence, the distribution from which households draw the first shock of their working lives must be chosen. If this first shock is assumed to be drawn from the stationary distribution of $s \in {\mathcal {E}}$, which is denoted by $\gamma ^{*}_{{\mathcal {E}}}$, then the intergenerational correlation of earnings will be very small. In contrast, if it is assumed that every working-age household inherits the endowment of efficiency labor units that its predecessor had at the end of its working life, then the intergenerational correlation of earnings will be relatively large. Since this correlation between generations usually lies between these two extremes, an additional parameter, $\phi _1$, is needed to act as a weight that averages between a matrix with $\gamma ^{*}_{{\mathcal {E}}}$ in every row, denoted by $\Gamma ^{*}_{{\mathcal {R}}{\mathcal {E}}}$, and the identity matrix, ${\mathbf {I}}$. Intuitively, the role played by this parameter is to shift the probability mass of $\Gamma ^{*}_{{\mathcal {R}}{\mathcal {E}}}$ toward its diagonal.

*Life cycle profile of income* The process driving the transition between retirement and working-age states, i.e., transition submatrix $\Gamma _{{\mathcal {R}}{\mathcal {E}}}$, should also reflect the existing earnings ability gap between new entrants and senior workers. Following the procedure presented in Castañeda et al. (2003), in order to determine such differential, the distribution from which households draw the first shock of their working lives must be chosen. If it is assumed that every new entrant starts its working stage with a shock drawn from $\gamma ^{*}_{{\mathcal {E}}}$, then the household earnings will be independent of household age. In contrast, if every household starts its working life with the smallest endowment of efficiency labor units, then the household earnings will grow too fast with household age. Since the earnings ability is parsimoniously increasing in household age, i.e., the earnings ability depends on household age, an additional parameter, $\phi _2$, is needed to act as a weight that averages between $\Gamma ^{*}_{{\mathcal {R}}{\mathcal {E}}}$ and a matrix with a unity vector in its first column and zeros elsewhere. Intuitively, the role played by this second parameter is to shift the probability mass of $\Gamma ^{*}_{{\mathcal {R}}{\mathcal {E}}}$ toward its first column.

A detailed description of the mass shifting procedure can be found in Sect. A.1 of “Appendix.” Unfortunately, the effects of $\phi _1$ and $\phi _2$ on the two magnitudes of interest previously mentioned are of opposite sign. Consequently, the modeling strategy to attain a pair of values for these parameters that induces magnitudes similar to those observed in the Spanish economy may be very parsimonious.

To keep the dimension of the process *s* as small as possible while still being able to achieve the calibration targets, a value of $J=4$ is chosen. This means that $J^2+J+4=24$ parameters need to be chosen. Note that $\Gamma _{{\mathcal {S}}\mathcal {S'}}$ has not yet been imposed to be a Markov matrix. When this is done, the number of free parameters is reduced to 20.

### Preferences and production possibilities

Households are assumed to derive utility from consumption, $c_t\ge 0$, and from non-market uses of their disposable time. They also care about utility of their descendents as if it were their own utility. Consequently, households’ preferences can be characterized by the following standard expected utility function:

2 $$\begin{aligned} E_{0} \left[ \sum _{t=0}^{\propto } \beta ^{t} u\left( c_{t,}\ell -h_{t}\right) |s_{0} \right] , \end{aligned}$$

where the function *u* is continuous and strictly concave in both arguments, $0<\beta <1$ is the subjective-time discount factor, $\ell$ is the endowment of productive disposable time, and $0\le h_t\le \ell$ is the labor choice. Consequently, $\ell -h_t$ is the amount of time allocated to non-market activities by the households. Note that retirees do not work, and consequently, they derive a constant utility from non-market activities.

The functional form chosen for the households’ common utility function is given by the following expression:

3 $$\begin{aligned} u(c,h)=\frac{c^{1-\sigma }}{1-\sigma }+\chi \frac{(\ell -h)^{1-\varphi }}{1-\varphi } \end{aligned}$$

where $\sigma$ denotes the curvature of consumption, $\varphi$ stands for the curvature of leisure (indirectly, the curvature of work) and the parameter $\chi$ controls the disutility from work. With this utility function, the intertemporal elasticity of substitution of consumption is given by $\frac{1}{\sigma }$, the intertemporal elasticity of substitution of leisure by $\frac{1}{\varphi }$ and the intertemporal elasticity of substitution of labor (i.e., the Frisch elasticity of labor supply) by $\frac{(\ell -h)}{\varphi \cdot h}$. This particular choice is widely used in this literature framework and is made because, as argued in Castañeda et al. (2003), the households in the model economy face very large changes in productivity, which, under standard nonseparable preferences, would result in extremely large variations of hours allocated to market activities. Aiming to avoid this, a more flexible functional form that is additively separable in consumption and leisure and that allows for different curvatures on these two variables is chosen. It implies that, to identify the households’ preferences, five parameters (the four that identify the utility function and the subjective time discount factor) must be chosen.

On the other hand, as far as production possibilities are concerned, an aggregate product definition, $Y_t$, that depends on aggregate capital, $K_t$, and aggregate labor, $L_t$, is chosen. Note that aggregate capital is obtained by aggregating the wealth (asset position) of every household, and aggregate labor input is obtained by aggregating the efficiency labor units supplied by every household. The aggregate production function, $Y_t=f(K_t,L_t)$, exhibits constant returns to scale. Therefore, the choice for the particular functional form here is the Cobb–Douglas production function $Y_t=A_t K^{\alpha }_t L^{1-\alpha }_t$, where $\alpha$ is the capital share and the total factor productivity, $A_t$, is normalized to 1. Further, capital is assumed to depreciate geometrically at a constant rate, $\delta$, and *r* and *w* are used to denote the prices of capital and of the efficiency units of labor before all taxes.^5^ Therefore, to depict the aggregate technology, the values of two parameters, $\alpha$ and $\delta$, must be chosen.

### Government sector

The government in the model economy taxes household income (from capital and from labor) and it uses the proceeds of taxation to make real transfers to retirees and to finance its consumption. Income taxes are described by the function $\tau (y_t)$, where $y_t$ denotes the household income. Public transfers to retirees are described by the function $\omega (s_t)$.

Social security in this model economy takes the form of transfers to retirees that do not depend on past contributions made by households, i.e., pensions are assumed to be fully redistributive.^6^ This is done out of computational convenience. These public pensions provide the non-working households with an insurance mechanism against the risk of living for too long; therefore, it reduces their incentives to save or accumulate assets for precautionary motives, which makes it easier to replicate the fraction of households that own very few or zero assets. This setup allows for matching the size of average public retirement pensions paid in Spain, but it qualifies the precision of the analysis in two ways. First, the overall amount of idiosyncratic risk in the model economy diminishes because the labor market history does not condition the retirement benefits. Second, it abstracts from a potentially important reason to work, since in real-world economies increasing the labor effort entitles the households to receive larger pension benefits.^7^

Therefore, a government policy rule in this model economy is a specification of $\{\tau (y_t),\,\omega (s_t)\}$ and of a process on government consumption, ${G_t}$. Since the government is running a budget balance scheme in every period, these policies must satisfy the condition $G_t+Tr_t=T_t$, where $Tr_t$ and $T_t$ denote aggregate transfers and aggregate tax revenues, respectively.

The household income taxes in the model economy are described by the function:

4 $$\begin{aligned} \tau (y)=\left[ y-\lambda y^{1-\tau }\right] +\kappa y \end{aligned}$$

The term within brackets is the tax function chosen by Heathcote et al. (2017) (HSV hereafter) to analyze the optimal progressivity of the tax system. Since this is the main purpose of this study, it justifies the election of such tax function for the model economy. More precisely, in this specification, $\lambda$ determines the average taxes while $\tau$ determines the progressivity. When $\tau =0$, taxes are flat and equal to $(1-\lambda )y$. When $\tau >0$, taxes are positive, and higher levels of $\tau$ imply a greater degree of progressivity (the marginal rates exceed the average rates). When $\tau <0$, the tax system is regressive (households with a nonnegative income would obtain a net transfer from the government). This specification has been already used by García-Miralles et al. (2019) to replicate the Spanish income tax system, and they show that the HSV specification matches almost exactly the effective average tax rates for the Spanish households.

The last term, $\kappa y$ is added to this tax function because the Spanish government obtains tax revenues from many other sources (property, estate, consumption, and excise taxes, among others), and this model economy abstracts from these tax sources. This choice implies that, in the model economy, it is assumed that all sources of tax revenues are proportional to income, which is equivalent to say that the government uses a proportional income tax to collect all the non-income tax revenues levied by the Spanish government. This type of augment of the tax function with a proportional term is widely used in the literature (e.g., see Castañeda et al. (2003) and Díaz-Giménez and Pijoan-Mas (2019)). Including this linear specification for remaining taxes could act as a consumption tax. This makes the choice of optimal progressivity more robust, since if consumption tax is not taken into account the optimal progressivity that the model finds can vary significantly, as argued by Guner et al. (2020). Therefore, to specify the model economy household income tax function, a total of three parameter values must be chosen.

### Market arrangements

In this model economy, there are no insurance markets for the household-specific shock. If insurance markets were allowed to operate this economy, the model economy would collapse to a standard representative agent model. Instead, to buffer their streams of consumption against the shocks, households in the model economy can accumulate wealth in the form of real capital, $a_t\in {\mathcal {A}}$. The lower bound of the compact set ${\mathcal {A}}$ can be interpreted as a form of liquidity constraints, or as a solvency requirement (preventing households that derive utility from leisure from going bankrupt).^8^ Finally, firms are assumed to rent factors of production from households in competitive spot markets, which implies that factor prices are given by the corresponding marginal productivities. This means that the interest rate, *r*, is equal to $\alpha K^{\alpha -1}L^{1-\alpha }-\delta$, and the wage, *w*, is equal to $(1-\alpha )(\frac{r+\delta }{\alpha })^{\frac{\alpha }{\alpha -1}}$.

### Households’ decision problem

The individual state variables are the realization of the household-specific shock, *s*, and the value of the asset holdings, *a*. The Bellman equation of the household decision problem is as follows:^9^

5 $$\begin{aligned}&\displaystyle v(a,s)\,=\,\max _{c,a',h}\,u(c,\ell -h)+\beta \,\sum _{s \in {\mathcal {S}}}\,\Gamma _{{\mathcal {S}}\mathcal {S'}}\,v(a',s') \end{aligned}$$

6 $$\begin{aligned}&s.t. \quad c+a'= y-\tau (y)+a \end{aligned}$$

7 $$\begin{aligned}&\quad \quad y = ar+e(s)hw+\omega (s) \end{aligned}$$

8 $$\begin{aligned}&\quad \quad \tau (y) = \left[ y-\lambda y^{1-\tau }\right] +\kappa y \end{aligned}$$

9 $$\begin{aligned}&\quad \quad c\ge 0 a'\in {\mathcal {A}} 0\le h\le \ell \end{aligned}$$

where *v* is the households’ common value function. Note that household income, *y*, includes three terms: capital income, *ar*, which can be earned by every household, labor income, *e*(*s*)*hw*, and retirement pensions, $\omega (s)$. Recall that $e(s)=0$ when $s\in {\mathcal {R}}$ and $\omega (s)=0$ when $s\in {\mathcal {E}}$. It is assumed that every household inherits the estate of the previous member of its dynasty at the beginning of the first period of its working life. More precisely, it is assumed that when a retiree exits the economy, it does so after that period’s consumption and savings have taken place. Then, at the beginning of the next period, the deceased household’s estate is liquidated and transmitted to the offspring.^10^ Here is the key that the asset choice for next period, $a'$, that an agent makes at *t* is the household’s stock wealth of its predecessor at the end of period *t* in the case that the predecessor is not going to be in the economy at period $t+1$. The household policy that solves this problem is a set of functions that map the individual state into the optimal choices for consumption, end-of-period savings, and labor hours. This policy is denoted by $\{c(a,s),\, a'(a,s),\, h(a,s)\}$.

### Equilibrium

Each period the economy-wide state is a probability measure of households, $x_t$, defined over an appropriate family of subsets of $\{{\mathcal {S}} \times {\mathcal {A}}\}$ that counts the households of each type, and that is denoted by ${\mathcal {B}}$. In the steady state, this measure is time-invariant, even though the individual state variables and the decisions of the individual households change from one period to the next one, as shown by Huggett (1993).

*Definition* A steady-state equilibrium for this economy is a household value function, *v*(*a*, *s*); a household policy, $\{c(a,s),\, a'(a,s),\, h(a,s)\}$; a government policy, $\{\tau (y),\,\omega (s),\,G\}$; a stationary probability measure of households, *x*; factor prices, (*r*, *w*); and macroeconomic aggregates, $\{K,\,L,\,T,\,Tr\}$, such that:

(i) Given factor prices and the government policy, the household value function and the household policy solve the households’ decision problem described in Eqs. (5)–(9).
(ii) Firms behave as competitive maximizers. That is, their decisions imply that factor prices are factor marginal productivities $r=f_K(K,L)-\delta$ and $w=f_L(K,L)$.
(iii) Factor inputs, tax revenues, and transfers are obtained by aggregating over households:
  $$\begin{aligned} K=\int \,a\,\mathrm {d}x;\;\;\; L=\int \,h(a,s)\,e(s)\,\mathrm {d}x;\;\;\; Tr=\int \,\omega (s)\,\mathrm {d}x;\;\;\; T=\int \,\tau (y)\,\mathrm {d}x \end{aligned}$$
  Every integral in the four definitions above is defined over the state space $\{{\mathcal {S}} \times {\mathcal {A}}\}$.
(iv) The goods market clears:
  $$\begin{aligned} \int \,\bigg \{c(a,s)+a'(a,s)\bigg \}\,\mathrm {d}x+G=f(K,L)+(1-\delta )K \end{aligned}$$
(v) The government budget constraint is satisfied: $G+Tr=T$.
(vi) The measure of households (i.e., the aggregate state variable) is stationary:
  $$\begin{aligned} x(B)=\int _{B}\,\bigg \{\int _{{\mathcal {S}} \times {\mathcal {A}}}\,[\xi _{_{a'(a,s)\in {\mathcal {A}}}}\xi _{_{s\in {\mathcal {S}}}}]\,\Gamma _{{\mathcal {S}}\mathcal {S'}}\,\mathrm {d}x\bigg \}\,\mathrm {d}a'\mathrm {d}s' \end{aligned}$$
  for all $B\in {\mathcal {B}}$, where $\xi$ is the indicator function that takes value 1 if some households choose certain level of assets and belong to certain shock group and 0 otherwise. This equation is aimed to count the households and to observe whether their asset holdings distribution is stationary. The procedure used to compute this equilibrium is in Sect. C.4 of “Appendix.”

## Calibration

The model economy is characterized by 36 parameters: 5 for preferences, 2 for production technology, 4 for government policy, and 25 for the joint process on the age of the households and on the endowments of efficiency labor units (implied by the choice of $J=4$ possible states during the working-age or the retiree phase of the life cycle). A full list of the parameters can be shown in Sect. B of “Appendix.”

To depict the values of these parameters, 37 calibration targets (T henceforth) are needed. Of these targets, 7 are normalization conditions (NC henceforth) that identify 7 parameters, and the remaining 30 are statistics that describe relevant features of the Spanish economy. Therefore, 30 target values describing the Spanish economy are needed to identify 29 parameters. Eight of these 30 calibration targets uniquely determine the value of 8 model economy parameters. These targets will be noted as direct identification (DI henceforth). To determine the values of the remaining 21 parameters, a system of 22 nonlinear equations must be solved. This system results from equating the values of 22 model economy statistics to their empirical analogues in the Spanish economy. The method of simulated moments (MSM henceforth) is proposed to solve this system. With respect to this method, it is important to recall that each parameter being calibrated by the MSM is intended to match one target of the remaining 22, but in practice a change in one parameter could induce changes in each of the 22 targeted model statistics. It means that there is necessarily no one-to-one correspondence between parameters and targets in the MSM system. In this sense, 22 target values are intended to calibrate 21 parameters. The remainder of this section is devoted to explain in a detailed way how each parameter of the model is identified and which parameters being calibrated by the MSM are intended to match which targets. The details of the procedure used to solve this system can be found in Sect. C.3 of “Appendix.”

*Model period* One important decision to be made is the length of the model period. It is set to be equal to one year since this is also the length of a tax period in Spain. Further, one year is also the length of the data collection period of the administrative panel dataset of tax returns used in this analysis. Finally, 2015 is chosen as the calibration year, as it is the last year with data availability of this particular data source. This means that the most updated estimate of the progressivity of the personal income tax at the household level depicts the situation in 2015.

*Normalization conditions* The household endowment of disposable time is an arbitrary constant and is chosen to be $\ell = 3.2$ [T1-NC (Target 1, Normalization Condition)], as standard in the related literature.^11^ The possible states in which a household can stay during its working-life or retirement are $J=4$ [T2-NC]. It means that the possible states of endowment of efficiency labor units will be 8: $\left[ e(1),e(2),e(4),e(5),e(6),e(7),e(8)\right]$. Recall that the endowment of efficiency labor units is zero for retired households, i.e., $e(s)=0$ for $s\in {5,6,7,8}$. This basically implies that the endowment vector of 8 possible states is characterized by 4 parameters (those related to working-age agents). In addition, the endowment of efficiency labor units of the least productive households is normalized to be $e(1)=1$ [T3-NC]. Finally, since matrix $\Gamma$ is a Markov matrix, its rows must add up to one. This property imposes four additional normalization conditions on the rows of $\Gamma _{\mathcal {EE}}$ [T4-NC to T7-NC]. The assumptions about the structure of the matrix $\Gamma$ imply that once submatrix $\Gamma _{\mathcal {EE}}$ has been appropriately normalized, every row of $\Gamma$ adds up to one without imposing additional restrictions. In this regard, the calibration algorithm normalizes the diagonal elements of $\Gamma _{\mathcal {EE}}$, i.e., $\Gamma _{\mathcal {EE}_{1,1}}$, $\Gamma _{\mathcal {EE}_{2,2}}$, $\Gamma _{\mathcal {EE}_{3,3}}$, and $\Gamma _{\mathcal {EE}_{4,4}}$. For instance, the calibration algorithm calculates the first diagonal element of $\Gamma _{\mathcal {EE}}$ as $\Gamma _{\mathcal {EE}_{1,1}}=1-\Gamma _{\mathcal {EE}_{1,2}}-\Gamma _{\mathcal {EE}_{1,3}}-\Gamma _{\mathcal {EE}_{1,4}}-p_{r}$, and analogously for each of the first four rows of $\Gamma$. The normalization condition could be placed in a different element of each row of the submatrix $\Gamma _{\mathcal {EE}}$, but the diagonal elements are chosen to be normalized out of computational convenience since it is known beforehand that most of the weight of the transition submatrix between working states is on the diagonal. Note that normalizing the diagonal element of $\Gamma _{\mathcal {EE}}$ the algorithm is less likely to deliver a negative value of it, since the rest of parameters involved in the normalization of the row ($\Gamma _{\mathcal {EE}_{1,2}}$, $\Gamma _{\mathcal {EE}_{1,3}}$, $\Gamma _{\mathcal {EE}_{1,4}}$, and $p_{r}$) normally take values close to zero. Thus in terms of maintaining the constraints on $\Gamma _{\mathcal {EE}}$ (all elements being between zero and one and rows summing up to one) required for it to be a transition matrix, the algorithm is less likely to be throwing out parameter vectors when estimating the calibrated values because they failed to meet these constraints.

### Macroeconomic aggregates and demographic targets

*Ratios* The target value for the capital-to-output ratio, *K*/*Y*, is 4.25 [T16-MSM], the capital income share, $\alpha$, is 0.48 [T8-DI], and the target value for the investment-to-output ratio, *I*/*Y*, is $21.94\%$ [T9-DI]. The target value for the capital- to-output ratio is obtained by dividing 247,523€, which was the average household net wealth in Spain in the calibration reference year according to the Bank of Spain (2017, 2019), by 58,230€, which was per household gross domestic product in Spain in the calibration reference year according to Eurostat (2020b, 2020c) and the Spanish National Institute of Statistics (2016) (*INE*, its acronym in Spanish).^12^ The parameter controlling for the subjective time discount factor, $\beta$, is calibrated to match this capital-to output-ratio. The target value for the capital income share is obtained by subtracting the labor income share from a total measure of 1. The value for the labor income share was 0.52 in Spain in the calibration reference year, according to data from EU KLEMS (2020). Thus the capital income share is directly identified as $\alpha =0.48$. To calculate the value of the target for *I*/*Y*, investment is defined as the sum of gross private fixed domestic investment, change in business inventories, and 75% of the private consumption expenditures in consumer durables using data for the calibration reference year from the Spanish National Institute of Statistics (2020).^13^ Further, note that the rate of depreciation of capital, $\delta$, follows immediately from $\delta =I/K$ in stationary general equilibrium, hence it is directly identified as $\delta =(I/Y)/(K/Y)=0.0516$. These choices amount to 3 targets.

*Allocation of time and consumption* General equilibrium heterogeneous agents models are built to asses counterfactual economies from both aggregate and individual perspective. To calibrate the model economy in the individual perspective, the intertemporal elasticity of substitution of consumption, determined by $\sigma$, the disutility from work, determined by $\chi$, and the Frisch elasticity of labor supply, determined by $\varphi$, are crucial parameters.

For the curvature of consumption, as usual in many papers, a value of $\sigma =1.5$ is chosen [T10-DI]. It induces a elasticity of intertemporal substitution of consumption of $\frac{1}{\sigma }=\frac{1}{1.5}=0,66$.^14^ The value chosen for $\sigma$ falls within the range (1-3) that is standard in the literature for the curvature of consumption. For example, the calibration–estimation exercise by Pijoan-Mas (2006) reports a value of 1.46, Pijoan-Mas and González Torrabadella (2006) encounter a value of 1.23, and Heathcote et al. (2010) finds a value of 1.44. In addition, Castañeda et al. (2003) and Díaz-Giménez and Pijoan-Mas (2019), research works that employ an utility function similar to the one herein presented, also identify directly the curvature of consumption, $\sigma$, as 1.5. This choice gives one additional target.

The parameter of the utility function controlling for the relative share of consumption and leisure, $\chi$, is calibrated by the MSM so as to match average share of disposable time allocated to market activities by the households. This statistic is targeted to be $H/\ell =30.83$ percent [T17-MSM]. The choice is based on the average daily length of time devoted to paid work by working agents in Spain in the calibration reference year according to the Spanish National Institute of Statistics (2011). It gives one more target. On the other hand, as it will be explained below, the parameter of the curvature of leisure (implying the Frisch elasticity of labor supply), $\varphi$, is selected within the range of values considered in the literature [T11-DI]. In this sense, some restrictions are placed in the values that the curvature of leisure, $\varphi$, could take, which accounts for one additional target. The parameter $\varphi$ is particularly important in the model, as it determines the elasticity of the labor supply and thus shapes the response of the labor supply to tax changes. A higher (lower) Frisch elasticity of labor supply will make the households react more (less) to tax changes. But there is still a lot of controversy in the literature about how large this elasticity should be. The early estimates of MaCurdy (1981), Altonji (1986), and Blundell and MaCurdy (1999) indicated that the labor supply elasticity was very small (much smaller than 0.5). However, these estimates are typically obtained for prime aged male actively engaged in the labor market, while elasticities for females are much larger, as shown by Kumar (2005). Furthermore, as pointed out by Pijoan-Mas and González Torrabadella (2006), once one also considers the extensive margin the estimates for the elasticity might even be larger. For example, Nobel laureate Prescott (2004) defends a Frisch elasticity of 1.50 for the labor supply. More specifically, by focusing on research works that are similar to the one presented in this paper and that try to evaluate fiscal reforms in the Spanish economy through macro- and micro-models, one can observe that the disparity in the values of the Frisch elasticity of labor supply is also a common element. For instance, Guner et al. (2020) set the Frisch elasticity of labor supply to 0.5, which implies a curvature of leisure, $\varphi$, equal to 4.25 (when measured at the average amount of hours worked). On the other hand, Pijoan-Mas and González Torrabadella (2006) and Díaz-Giménez and Pijoan-Mas (2019) estimate in their model economies that the elasticity of the intertemporal substitution of labor is around 1.8, which would translate into curvatures of consumption of $\varphi =1.15$. Therefore, the parameter $\varphi$ will be calibrated in the model economy such that it falls within the range observed in the literature applied to the Spanish economy, i.e., Frisch elasticities of labor supply ranging from 0.5 up to 1.8. Making use of this strategy, as it will be shown in Table 2 of Sect. 3.4, the value of the curvature of leisure will be given by $\varphi =2.65$. It would imply that the model economy would present a Frisch elasticity of labor supply equal to $\frac{(\ell -h)}{\varphi \cdot h}=\frac{(3.2-0.99)}{2.65 \cdot 0.99}=0.85$ (when evaluated at its average). This value is in line with Imai and Keane (2004), Domeij and Flodén (2006) and Chetty (2012), who argue that a value slightly greater than 0.5 is reasonable. In addition, the labor supply of the model economy will be very similar to the one encountered by Pijoan-Mas (2006). It should be also noted that a labor supply elasticity of 0.85 falls in the middle of the value spectrum found in the literature devoted to the Spanish economy, which reinforces the strategy chosen to calibrate the parameter $\varphi$.

*Age structure of the population* The expected duration of working-lives in Spain is targeted to be 35 years [T12-DI], according to Eurostat (2020a). The expected duration of retirement in Spain is targeted to be 22.8 years [T13-DI], according to the OECD (2015, 2017).^15^ These values serve as two more targets that directly identify the probability of retiring, $p_{r}=\frac{1}{35}=0.0286$, and the probability of surviving, $p_{s}=1-\frac{1}{22.8}=0.9561$, respectively.

*Life cycle profile of income* To replicate the life cycle profile of income in Spain in the model economy, the ratio of the average annual wage of agents between ages 45 and 49 to that of agents between ages 25 and 29 is the target value [T18-MSM]. The transition process between retirement and working-age states will be therefore calibrated in such a way that will reflect the observed differences in earnings ability between new entrants and senior workers. In 2015, the value of this statistic in Spain was 1.56, according to the Spanish National Institute of Statistics (2017). This provides an additional target which is intended to calibrate by the MSM the controller of the life cycle earnings profile, i.e., the parameter $\phi _1$.

*Intergenerational transmission of earnings ability* To replicate the intergenerational correlation of income encountered in Spain in the model economy, the target is the correlation between the average income of one generation and the average income of its immediate descendents. Llaneras et al. (2020), in their *Atlas de Oportunidades* project, developed a database at the household and individual level originating from the Spanish State Agency of Tax Administration (AEAT, its Spanish acronym). In this database, 2.7 million young people in 2016 can be followed and for each of them their current income and the income of their parents’ home (in the period 1980–1990) can be obtained. This database is the most updated attempt to study the social mobility in Spain and the intergenerational income correlation between sons and daughters with respect to their parents’ income. Making use of such prominent database, the intergenerational correlation of income at the household level in Spain is estimated to be 0.50 [T19-MSM].^16^ This translates into one more target value which is intended to calibrate by the MSM the controller of the intergenerational persistence of earnings, i.e., the parameter $\phi _2$.

### Government policy

The parameters of the model economy household income tax are chosen so that the government collect the total tax revenues observed in the Spanish economy, which were 33.63% of the GDP in Spain in the calibration reference year, according to the OECD (2020). In the model economy, these revenues must be entirely spent by the government, i.e., the government of the model economy must balance its budget. This means that the output shares of government consumption, *G*/*Y*, and government transfers, *Tr*/*Y* (the two government expenditure items in this model economy), are required to add up to 33.63%, which was the GDP share of total tax revenues, *T*/*Y*, in Spain in the calibration reference year, as previously mentioned. The target value for the transfers-to-output ratio in the model economy is 11.36% [T20-MSM], which corresponds to the GDP share accounted for by social security contributions in Spain in the calibration reference year, according to the OECD (2020). This value is chosen so because the retired agents in the model economy receive a lump-sum transfer that, once it is aggregated, must be the same as the social security contributions that were paid by the working-age households. This choice means that the residual share for government expenditures to GDP amounts to 22.27% ($=33.63\%-11.36\%$) [T21-MSM], which is the target for the *G*/*Y* ratio in the model economy. This is consistent with World Bank (2020b) data for Spain for the calibration reference year, which present a general government final consumption expenditure (as % of GDP) of 22%.

The personal income tax function of the model economy is aimed to mimic the progressivity of the Spanish effective personal income taxes. This objective is achieved by shaping the tax function with an augmented version of the HSV specification, as it was similarly done by García-Miralles et al. (2019). The specification used in the present analysis was introduced in Eq. 4. To identify it, the values of parameters $\lambda$, $\tau$, and $\kappa$ must be chosen.

**Fig. 1:**
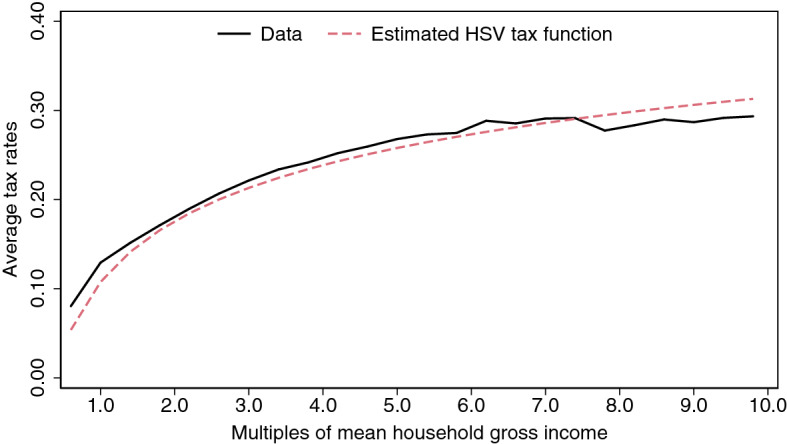
Fitting of the HSV specification to the data

Following the estimation strategy proposed by García-Miralles et al. (2019), the parameters $\lambda$ and $\tau$ can be directly estimated using an administrative panel dataset containing a (stratified) random sample of tax returns.^17^ This dataset is provided by the Spanish State Agency of Tax Administration (2019) and contains a very detailed account of income from different sources, tax benefits, tax liabilities, and sociodemographic characteristics for a sample that accounts for a 14% of the population.^18^ The last update of this panel dataset at the household level covers the tax returns in 2015. Hence, the estimated progressivity of the tax system depicts the situation in that year. The estimation results in parameters $\lambda$ and $\tau$ taking values 0.8924 and 0.1146 [T14-DI and T15-DI], respectively. These two targets result from directly imposing that the shape of the model economy’s tax function coincides with the shape of the estimated HSV specification. As it can be shown in Fig. 1, the fitting of the estimated HSV tax function to the 2015 household data is very precise, which justifies the choice of this specification for the model economy.

Finally, we just need to choose the values of the parameter $\omega$, which stands for the lump-sum transfers to retirees, and the remaining parameter of the households’ income tax function of this model economy that has not been already chosen, $\kappa$. More precisely, $\omega$ is chosen so as to achieve that the model economy transfers-to-output ratio, *Tr*/*Y*, mimics the value observed in the Spanish economy. On the other hand, the value of $\kappa$ is chosen so that the government expenditures-to-output ratio in the model economy, *G*/*Y*, matches that observed amount in the Spanish economy. Note that the government in this model economy only allocates its spending on social security transfers and government spending. Then, because the two above parameters are chosen to match these two targets, it can also be said that the parameter $\kappa$ is chosen so as to match the ratio of total tax revenues to output, *T*/*Y*, observed in the Spanish economy. This is equivalent to stating that the value of $\kappa$ is selected such that the government in the model economy runs a budget balance policy in equilibrium, i.e., $G+Tr=T$. Indeed, the value of $\kappa$ is only completely calibrated when the general equilibrium of the economy is found, since such parameter acts as price solver of the general equilibrium by ensuring that the government runs a budget balance policy. These choices, in conjunction with those that mimic the shape of the model economy’s tax function, represent 4 more targets.

### Distributions of income and wealth

The aforementioned conditions specify a total of 21 targets (7 normalizations and 14 target values observed in the Spanish economy) out of a total of 37 targets. To solve this model economy, one must choose the value of 36 parameters. Therefore, 15 additional parameters must be identified. These parameters will be calibrated by the MSM (jointly with the previously mentioned ones that will also enter the MSM calibration strategy) so as to match 16 additional targets covering the Spanish distributions of income and wealth are added: (i) the Gini coefficients [T22-MSM and T23-MSM]; (ii) the income and wealth shares of the households concentrated between percentiles 1 to 40, 40 to 60, 60 to 80, and 80 to 100 [T24-MSM to T31-MSM]; and (iii) the income and wealth shares of the households concentrated between percentiles 90 to 95, 95 to 99, and the top 1% [T32-MSM to T-37-MSM]. The same calibration procedure can be found in Castañeda et al. (2003) and Díaz-Giménez and Pijoan-Mas (2019). These 16 calibration targets related to the wealth and income distributions are intended to estimate by means of the MSM 3 out of 4 endowments of efficiency labor units (*e*(1), *e*(2), and *e*(3), since *e*(1) is normalized to be 1) and the off-diagonal elements (12 elements) of the submatrix driving the transition between working states, $\Gamma _{\mathcal {EE}}$. (The diagonal elements originate from a normalization procedure, as explained before.)

For wealth distributional statistics, the 2015 household data to get the target statistics come from a linear interpolation between statistics derived from the 2014 and the 2017 waves of the Spanish Survey of Household Finances, a very influential survey conducted by the Bank of Spain (2017, 2019) detailing the household balance sheets and their wealth positions. Since the 2017 wave of the Spanish Survey of Household Finances is not completely available yet, some information for such year is directly extracted from the 2017 wave of European version of it, the Household Finance and Consumption Survey, which is conducted by the European Central Bank (2020). As for the income distributional statistics, the 2015 household data to get the target statistics come from the aforementioned administrative tax data provided by the Spanish State Agency of Tax Administration (2019).

### Calibration outcomes

This subsection presents the complete parametrization of the model economy. The values of the 36 parameters are presented here in two tables. The calibrated parameters characterizing the stochastic process of the endowment of efficiency labor units are reported in Table 1, while the rest of the calibrated parameters characterizing the model economy are shown in Table 2. Finally, Table 3 displays the fitness of the model economy, where the success of the parametrization to replicate key selected characteristics of the Spanish economy can be observed.

*Stochastic process of endowment of efficiency labor units* With the exception of the first endowment, *e*(1), and the diagonal elements of the submatrix $\Gamma _{\mathcal {EE}}$, which are determined by normalization, the parameters presented here are estimated/calibrated by means of the MSM in order to match the targets related to the wealth and income distributions presented in the previous Subsection. This means that, of the 20 parameters that this process has, 5 are identified through normalization and 15 through MSM estimation jointly with the rest of the model economy parameters that are also estimated by this procedure.

**Table 1:** Stochastic process for the endowment of efficiency labor units

	*e*(*s*)	$\gamma ^{*}_{{\mathcal {E}}}$	$\Gamma _{{\mathcal {E}}{\mathcal {E}}}$ from *s* to $s'$			
			$s'=1$	$s'=2$	$s'=3$	$s'=4$
$s=1$	1.00	15.17	89.58	10.36	0.01	0.05
$s=2$	2.71	65.15	2.42	96.54	1.03	0.01
$s=3$	7.80	18.39	0.01	3.60	96.34	0.04
$s=4$	90.00	1.28	0.01	1.73	0.01	98.25

*e*(*s*) denotes the relative endowment of efficiency labor units; $\gamma ^{*}_{{\mathcal {E}}}$ denotes the stationary distribution of working-age households; $\Gamma _{{\mathcal {E}}{\mathcal {E}}}$ denotes the transition probabilities of the process on the endowment of efficiency labor units for working-age households that are still workers one period later

**Table 2:** Parameter values for the baseline model economy

Parameters			Strategy	Target		
Definition		Value		Definition		Value
*Preferences*						
Subjective time discount factor	$\beta$	0.9551	MSM	Capital-to-output	${K}\big /{Y}$	4.25
Curvature of consumption	$\sigma$	1.5000	DI	Elasticity of consumption	${1}\big /{\sigma }$	${1}\big /{3}$
Curvature of leisure	$\varphi$	2.6500	DI	Frisch elasticity of labor supply	${(\ell -h)}\big /{(\varphi \cdot h)}$	0.85
Relative share of consumption and leisure	$\chi$	0.5002	MSM	Share of time devoted to work	${H}\big /{ell}$	30.83%
Endowment of discretionary time	$\ell$	3.2000	NC	Aggregate labor input	H	$\approx$1.00
*Technology*						
Capital income share	$\alpha$	0.4755	DI	Capital income share	${r\cdot K}\big /{Y}$	47.55%
Capital depreciation rate	$\delta$	0.0516	DI	Investment to output	${I}\big /{Y}$	21.94%
*Fiscal policy*						
Normalized transfer to retirees	$\omega$	3.2150	MSM	Transfers to output	${Tr}\big /{Y}$	11.36%
Average level of income taxation	$\lambda$	0.8924	DI	Estimated $\lambda$ in HSV	${\hat{\lambda }}$	0.8924
Progressivity of income taxation	$\tau$	0.1146	DI	Estimated $\tau$ in HSV	${\hat{\tau }}$	0.1146
Linear term on remaining taxes	$\kappa$	0.0524	MSM	Government exp. to output	${G}\big /{Y}$	22.27%
*Age and endowment process*						
Number of possible working/retirement states	J	4	NC	Standard in literature	–	–
Probability of retiring	$p_r$	0.0286	DI	Expected duration of working life	–	35 years
Probability of dying	$1-p_s$	0.0439	DI	Expected duration of retirement	–	22.77 years
Life cycle earnings profile	$\phi _1$	0.9999	MSM	Ratio old-to-young income	$\rho _{o,y}$	1.56
Intergenerational persistence of earnings	$\phi _2$	0.9715	MSM	Correlation between father and son	$\rho _{f,s}$	0.50

The process, as shown in Table 1, presents strong skewness, persistence of the shocks, fat right tail, and nonlinear dynamics. It is important not to take this process literally, since it is an approximation that represents everything that is not known about the model economy. The relative endowments of efficiency labor units are reported in the second column of Table 1 and the invariant measures of each type of working-age households are in the third column. The endowments of workers are calibrated such that the endowment of the least lucky (in terms of endowments of efficiency labor units) household is 1 (normalization). Then, it can be observed that the luckiest workers in the model economy are 90 times as lucky as the unluckiest ones. The stationary distribution shows that each period 15% of the workers are very unlucky and draw state $s=1$. The working agents are mainly concentrated around state $s=2$, with two out of three workers receiving this productivity shock each period. The transition probabilities between the working-age states are reported in the last four columns of the table. Here it can be noted that agents in states $s=3$, $s=4$, and especially $s=1$ have higher probability of moving to $s=2$ than to other possible working states. However, each period only one out of every 100 workers is extremely lucky and draws state $s=4$. Only very rarely workers whose current state is $s\ne 4$ will make a transition to state $s=4$. Finally, there is a group of households that are not that lucky in comparison with those households that draw state $s=4$, but they are not as unlucky as the ones who draw states $s=1$ or $s=2$. This group of households accounts for 18% of the workers drawing state $s=3$ each period. The concentration of the invariant distribution of working households in the first two states is typically higher in the literature devoted to match the characteristics of the US economy with this type of models, as it can be shown in Castañeda et al. (2003) and Díaz-Giménez and Pijoan-Mas (2019). Nevertheless, since the level of income inequality in Spain is much lower, this weight decreases in order to generate an income distribution with higher income-shares in the not-top percentiles.

Note that Table 1 only represents the submatrix $\Gamma _{\mathcal {EE}}$. This submatrix drives the transition of working households between working states. Recall from expression (1) that this particular submatrix is only the upper-left part of the overall transition matrix $\Gamma$, but there are many other possible transitions between working to non-working states and vice versa. The complete transition matrix between all possible states of the model economy, $\Gamma$, and the complete invariant distribution of households, $\gamma ^{*}$, are presented in Table 8, in Sect. A.2 of “Appendix.” This complete Markov Chain transition matrix is what features the stochastic process that jointly defines the age and the endowment of labor efficiency units of each household.

*Other parameter values* Every other parameter of the model economy is presented in Table 2. Jointly with the calibrated values for the parameters, it can be observed which calibration strategy was used to identify each of them. Among these parameters, 8 are directly identified to match some targets, 2 are normalization conditions, and 6 parameters were calibrated by solving a system of 22 nonlinear equations (by the MSM) jointly with those calibrated by the same method in the stochastic process of endowment of efficiency labor units. In addition, in case that a parameter would have been calibrated to match some target statistic of the Spanish economy, it is also shown in the last columns of the table. These last columns indicate to which target statistic each parameter points out.

**Table 3:** Baseline model economy (BE) and Spanish economy (Spain)

Macroeconomic and fiscal ratios								
Economy	*K*/*Y*	*I*/*Y*	*G*/*Y*	*T*/*Y*	*Tr*/*Y*	${H}\big /{ell}$	$\rho _{o,y}$	$\rho _{f,s}$
Spain	4.25	21.94	22.27	33.63	11.36	30.83	1.56	0.50
BE	4.26	22.00	22.27	33.51	11.24	30.78	1.53	0.50

Distributional statistics								
Economy	Gini	Percentiles (%)				Top groups (%)		
		$<40$	40-60	60-80	80-100	90-95	95-99	99-100
*The distribution of income (before all taxes and after transfers)*								
Spain	0.48	12.72	13.84	21.19	52.25	11.01	13.40	12.07
BE	0.45	14.72	13.72	21.32	50.24	10.85	13.35	13.57
*The distribution of wealth*								
Spain	0.68	3.62	9.65	18.11	68.62	12.93	19.79	20.27
BE	0.68	3.80	9.32	17.45	69.43	13.54	19.68	19.63

${H}\big /{ell}$ denotes the share of disposable time allocated to market activities; $\rho _{o,y}$ denotes the ratio of the average income of agents between ages 45 and 49 (old) to that of agents between ages 25 and 29 (young); $\rho _{f,s}$ denotes the correlation between the average income of one generation (fathers) and the average income of their immediate descendents (sons)

*Fitness of the model economy* The statistics that describe the main aggregate and distributional features of the Spanish and the baseline model economies are reported in Table 3. In this table, it can be noticed how well the selected parametrization matches the calibration targets. These results confirm that overall the model economy succeeds in replicating the most relevant features of the Spanish economy in much detail. This result is particularly promising since it delivers a very well suited benchmark model to evaluate several potential reforms of the tax and transfers system.

## Optimal progressivity

This study aims to analyze what the optimal progressivity level of the personal income tax in Spain would be. Namely, it makes a comparison between the calibrated baseline model economy with the current estimated level of progressivity ($\tau =0.1146$) and a grid of several alternative economies exhibiting different progressivity levels. In every alternative scenario, the progressivity parameter $\tau$ is updated to a different value and a new general equilibrium for each reformed model economy is calculated.

But how should the reform be designed? The literature has conventionally employed two types of criteria when evaluating potential tax reforms through theoretical models. On the one hand, the most common choice within this type of research has been to assess reforms that are revenue-neutral. That is, reforms in which a change in taxation leaves the government’s tax revenue levels unchanged, and therefore, the levels of government expenditure, *G*, and transfers, *Tr*. In contrast, another body of academic literature in this area has focused on tax reforms that maintain the same level of tax burden on the economy (i.e., on the income-average household). That is, the government must design a tax reform in such a way that the ratio of tax revenues to GDP, *T*/*Y*, remains constant. This is the approach chosen in the study herein presented. In this sense, this paper evaluates tax reforms that change the level of tax progressivity without changing the average tax rate (as measured from aggregate data). In addition, the design of the reform must respect the government’s spending structure. In other words, the reform must hold not only the *T*/*Y* ratio unchanged, but also the output shares of the transfers, *Tr*/*Y*, and of the government expenditure, *G*/*Y*. Being output, *Y*, a measure of the size of the economy, it would imply that for each potential size of the economy the government would offer the same relative amount of education, health, coverage to non-workers, pensions to retirees, etc.

In practice, it translates into every reformed economy being designed such that several conditions are satisfied: (i) the markets clear and (ii) the government runs a balanced-budget policy (iii) while keeping pensions and government expenditure shares of output fixed at baseline levels. Each reformed economy with a different progressivity level exhibits a new interest rate, *r*, and new wage, *w*, such that the goods and assets markets clear. This particular reform design implies that the government must choose in each reformed economy a new average level of taxes, $\lambda$, such that, with a certain progressivity level, $\tau$, it balances its budget and leaves the ratios $Tr/Y=11.24\%$ (varying transfers to retirees, $\omega$, so as to match this level) and $G/Y=22.27\%$ fixed at their baseline levels. It means that in every reformed economy, the combination of $\lambda$, $\tau$, and $\omega$ chosen by the government must induce the same output-share accounted for by personal income tax revenues as in the baseline economy, i.e., $T/Y=33.51\%$. A more involved explanation of the general equilibrium calculation in a reformed economy can be accessed in Sect. C.4.1 of “Appendix.”

After calculating a grid of several alternative economies exhibiting different progressivity levels, $\tau$, ranging from 0.00 to 0.50, the optimal reform of the personal income tax progressivity would be the one that maximizes the aggregate welfare gains. Note that economies with negative progressivity, $\tau <0$, are not here considered as they would induce a regressive income tax system, which exceeds the scope of the analysis. Further, economies with progressivity $\tau >0.5$ are not here reported because they always present enormous aggregate welfare losses and the focus of the analysis should be put in the reformed economies that are closer to the baseline economy and to the optimal one.

### Welfare changes and optimal progressivity level

Which economy with level of progressivity from $\tau =0.00$ to $\tau =0.50$ results in a steady state with higher aggregate welfare?^19^ Are these aggregate welfare changes mainly driven by the change in the tax scheme? Or do they principally arise from induced shifts in the distribution of households (distributional effects) or in the prices of the economy (general equilibrium effects)? How are these welfare changes distributed across households? A Benthamite social welfare function is used to answer these sort of concerns. The Benthamite social planner maximizes a welfare function that gives identical weights to every household in the economy. Consequently, when the utility function is concave, equal sharing is the welfare-maximizing allocation. It should be noted that the welfare outcomes here presented emerge from a comparison between the welfare derived from different steady state allocations. The present work remains silent about the transitions between these steady states.

#### Aggregate welfare changes and selection of the optimal progressivity level

To carry out the welfare comparisons, let $v_{BE}(a,s,\bigtriangleup )$ be the equilibrium value function of a household of type (*a*, *s*) in the baseline model economy, whose equilibrium consumption allocation is changed by a fraction $\bigtriangleup$ every period and whose leisure remains unchanged. Formally,

10 $$\begin{aligned} v_{BE}(a,s,\bigtriangleup )&=u(c_{BE}(a,s)(1+\bigtriangleup ),\ell -h_{BE}(a,s)) \nonumber \\&\quad +\beta \,\sum _{s \in {\mathcal {S}}}\,\Gamma _{{\mathcal {S}}\mathcal {S'}}\,v(a'_{BE}(a,s),s',\bigtriangleup ) \end{aligned}$$

where $c_{BE}(a,s)$, $h_{BE}(a,s)$, and $a'_{BE}(a,s)$ are the solutions to the households’ decision problem defined in expressions (5) to (9). Next, the welfare gain of living in the steady state of a reformed economy, $E_{\tau }$, for $\tau =\{0.00,\ldots , 0.50\}$, is defined as the fraction of additional consumption, $\bigtriangleup _{\tau }$, that must be given to, or taken away from, the households of the baseline economy so that the aggregate steady-state welfare in the reformed economy $E_{\tau }$ is the same as in the baseline economy $E_{BE}$. Formally, $\bigtriangleup _{\tau }$ is the solution to the following equation:

11 $$\begin{aligned} \int \,v_{BE}(a,s,\bigtriangleup )\mathrm {d}x_{BE}=\int \,v_{\tau }(a,s)\mathrm {d}x_{\tau }, \end{aligned}$$

where $v_{\tau }$ and $x_{\tau }$ are the equilibrium value function and the equilibrium stationary distribution of households in the reformed economy, $E_{\tau }$.

**Fig. 2:**
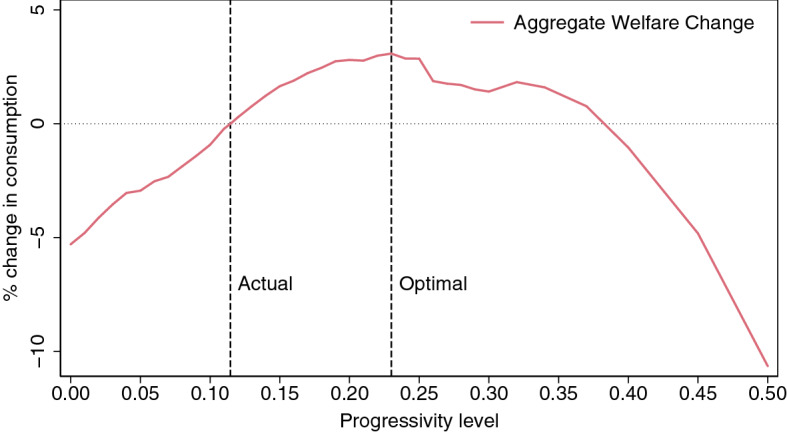
Aggregate welfare change

The aggregate welfare gains or losses associated with each progressivity level are depicted in Fig. 2. It can be here observed that any reform that increases the progressivity level from the current level (current level, baseline level, or “actual level” are used interchangeably), $\tau =0.1146$, up to levels of $\tau =0.37$ would result in aggregate welfare gains measured in percent changes of average household consumption. In this regard, the optimal reform of the progressivity level of the Spanish personal income tax would be encountered for $\tau =0.23$. An equivalent variation in consumption of 3.08% would be found in such optimal case. This means that, from a Benthamite perspective, the steady state generated by a reform of implementing a progressivity level of $\tau =0.23$ and increasing the average level of taxes accordingly up to $\lambda =1.18$ would be largely preferred to the steady state under the actual scenario (i.e., $\tau =0.1146$ and $\lambda =0.8924$). Indeed, the consumption of the average household of the model economy would need to be increased by 3.08% in every period and in every state for the social planner to be indifferent between the steady-state allocation implied by the actual progressivity level and the steady-state allocation that results from establishing a progressivity level of $\tau =0.23$ and increasing the average level of taxes accordingly up to $\lambda =1.18$.^20^ Therefore, it can be deduced that implementing levels of progressivity lower than $\tau =0.1146$ or higher than $\tau =0.37$ would generate losses of aggregate welfare according to this setup.

#### A decomposition of the aggregate welfare changes

To improve the understanding of the results from an aggregate welfare change perspective, it is useful to decompose the aggregate equivalent variation in consumption previously presented. In order to do so, two additional measures of equivalent variations in consumption are defined. First, a particular equivalent variation in consumption is computed so that it makes the households indifferent between the baseline economy, $E_{BE}$, and the optimally reformed economy, $E_{0.23}$, ignoring changes in the equilibrium distribution of households across value function levels. Let $\bigtriangleup _{0.23}^{a}$ be such variation, which is defined as follows:

12 $$\begin{aligned} \int \,v_{BE}(a,s,\bigtriangleup _{0.23}^{a};r_{BE},w_{BE})\mathrm {d}x_{BE}=\int \,v_{0.23}(a,s;r_{0.23},w_{0.23})\mathrm {d}x_{BE} \end{aligned}$$

Note that the aggregate welfare of such hypothetical economy is calculated using the equilibrium price vector of the optimally reformed economy, $(r_{0.23},w_{0.23})$, while the equilibrium stationary distribution is calculated using that of the baseline model economy, $x_{BE}$.

Second, another particular equivalent variation in consumption is computed so that it makes the households indifferent between the baseline model economy, $E_{BE}$, and the optimally reformed economy, $E_{0.23}$, ignoring both changes in the equilibrium distributions of households across value function levels and changes in the size of the economy. Let $\bigtriangleup _{0.23}^{b}$ be such variation, which is defined as follows:

13 $$\begin{aligned} \int \,v_{BE}(a,s,\bigtriangleup _{0.23}^{b};r_{BE},w_{BE})\mathrm {d}x_{BE}=\int \,v_{0.23}(a,s;r_{BE},w_{BE})\mathrm {d}x_{BE} \end{aligned}$$

Note that the aggregate welfare of such hypothetical economy is now calculated using both the equilibrium stationary distribution, $x_{BE}$, and the equilibrium price vector, $(r_{BE},w_{BE})$, of the baseline model economy.

These two additional equivalent variations in consumption allow for decomposing the total equivalent variation in consumption defined in expression (11) as follows:

14 $$\begin{aligned} \bigtriangleup _{0.23}=\underbrace{\bigtriangleup _{0.23}^{b}}_\text {(1)}+\underbrace{(\bigtriangleup _{0.23}^{a}-\bigtriangleup _{0.23}^{b})}_\text {(2)}+\underbrace{(\bigtriangleup _{0.23}-\bigtriangleup _{0.23}^{a})}_\text {(3)} \end{aligned}$$

The first term (1) of the right-hand side in Eq. (14) measures the aggregate welfare changes that are due to the reshuffling of resources between the households and ignores both general equilibrium effects of the optimal reform and changes in the distribution of households, i.e., it measures the welfare changes stemming from pure changes in the tax system. The second term (2) shows the added aggregate welfare change triggered by changes in equilibrium prices, i.e., it measures the general equilibrium effects of the optimal reform. The last term (3) measures the additional aggregate welfare change associated with changes in the equilibrium distribution of households across value function levels.

**Table 4:** Decomposition of aggregate welfare changes

Aggregate consumption equivalent variation	3.08%
Decomposition—contributions (in %) to the aggregate welfare change by changes in	
Tax system	121.38%
Equilibrium prices	$-\,19.25\%$
Equilibrium distribution	$-\,2.13\%$

Each contribution to the aggregate welfare change is computed by dividing the consumption equivalent variation from changes in each factor by the aggregate consumption equivalent variation. Adding up three contributions makes one hundred percent

The decomposition results are presented in Table 4. It is certainly interesting that most of the welfare gains are obtained by direct improvements in the tax system. It means that most of the aggregate welfare gains come from poorest households facing lower effective income tax rates and richest households affronting higher effective income tax rates. In contrast, general equilibrium effects of setting the optimally reformed economy and effects resulting from shifts in the equilibrium distribution of households across income levels show a welfare loss, but these losses are that small that together cannot overpass the welfare gains coming from the pure reform of the tax and transfers system, jointly resulting in aggregate welfare gains. The reform provokes a shrinkage in wages (*w* declining from 1.95 to 1.77 in the model) and an increment in the interest rate (*r* rising from 5.99 to 7.20% in the model), both reactions making the aggregate welfare change associated with equilibrium prices be negative. When it comes to welfare changes resulting from changes in the equilibrium distribution of households across income levels, these changes are negative, although they are almost zero. It means that there will be a larger mass of households at lower income levels. However, despite these effects, the change in aggregate welfare is largely positive, showing how powerful such reform of the tax system is. This shows and reinforces the optimality of raising the progressivity level of the personal income tax in terms of aggregate welfare.

#### Welfare changes by household types

The welfare gains and losses resulting from comparing the baseline model economy, $E_{BE}$, and the optimally reformed economy, $E_{0.23}$, are decomposed for different types of households in the spirit of Díaz-Giménez and Pijoan-Mas (2019). In the model economy, there are as many households as there are $\{a,s\}$ pairs in the individual state space. To calculate the welfare changes at each point in the state space, $\bigtriangleup _{0.23}(a,s)$, the following equation is solved.

15 $$\begin{aligned} v_{BE}(a,s,\bigtriangleup _{0.23}(a,s))=v_{0.23}(a,s) \end{aligned}$$

**Fig. 3:**
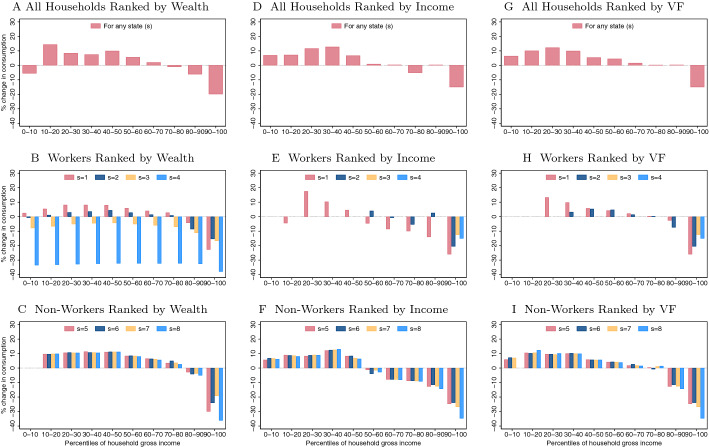
Welfare changes by type of household

The average of these individual welfare changes for various decile groups of households are reported in Fig. 3.

*Sorting by wealth* The households are here sorted by their asset holding positions and the welfare gains are computed as an average over every household (i.e., over every pair of states $\{a,s\}$) belonging to a certain group. The division of the wealth distribution in ten deciles leaves groups of households accounting for ten percentile points each. Looking at Panel A, ranking every household by wealth, it can be seen that households within percentile 10 and percentile 70 are those that benefit from the reform, being these welfare gains decreasing in wealth. Contrarily, households in the two top wealth deciles and, curiously, households in the lowest wealth decile are the ones that would experience welfare losses with the reform. But what are the driving forces behind these welfare changes by wealth decile? A deeper understanding of it could be learned from observing Panels B and C.

First, the positive welfare variation within the 2nd and the 7th wealth deciles could be mainly explained by the welfare gains experienced by (ii) non-working households and (ii) the least productive working households populating those deciles of the wealth distribution. The welfare changes of retired households (those receiving shocks $s=5$ to $s=8$) are reported in Panel C. Here it can be noticed that every non-working household located within percentiles 10 and 80 of the wealth distribution experiences welfare gains, with these gains decreasing when the household becomes wealthier. These retired households in this part of the wealth distribution benefit from the reform because their income (who is compounded by transfers, $\omega$, and returns from capital) is so low that is taxed at a lower rate due to a higher progressivity level. In addition, working households located within percentiles 10 and 80 of the wealth distribution and receiving the lowest endowments of efficiency labor units ($s=1$ and $s=2$) also experience welfare gains from the reform, as shown in Panel B. They particularly benefit from a more progressive tax system that levies less taxes on the lowest earnings. Although it is observed that working households receiving a large productivity shock ($s=3$ and especially $s=4$) are those who are most negatively affected by the reform (since they face a higher income tax rate due to a more progressive tax system), they only account for a small fraction of the household stationary distribution, thus resulting in aggregate positive welfare changes of working households populating the wealth distribution within its 10th and 80th percentiles.

Second, a plausible explanation for the welfare downturn faced by households encountered in the lowest wealth decile is that this particular decile, as shown in Panels B and C, is solely populated by working-age households with zero or very few assets. These working households, even though they could be very productive (receive shocks $s=3$ or $s=4$), are so wealth-poor that have to work more to be able to afford their consumption. These could be households at early stages of their labor market career who still did not have time to accumulate wealth. In this sense, their earned labor income (which is the only income they may have), and especially the labor income of the most productive agents (those receiving $s=3$ and $s=4$) with few assets, is taxed at a higher rate as progressivity increases, which makes them suffer welfare losses.

Finally, the welfare losses observed in the top 20% of the wealth distribution could be rationalized in the same way for both working and retired households. These households are so wealth-rich that their capital income gains will increase in a substantial proportion due to a higher return of capital, *r*. When the progressivity level rises, there is less capital accumulation for self-insurance or due to precautionary motives because the government runs a more redistributive income tax scheme that provides households with alternative insurance mechanisms against future income shocks. It results in an increase in the interest rate, *r*, which consequently will augment the capital income gains and thus the total personal income. This augmented income will be taxed at a so high tax rate (due to a higher progressivity level) that would lead to very important welfare losses of the top-wealth households. Further, this negative welfare effect through the capital income channel offsets the potential positive welfare effects that the least productive working households ($s=1$ and $s=2$) could find through a labor income being taxed at a lower rate.

*Sorting by income* In panel D, households are sorted by their income before all taxes and after transfers. The most benefited households are those at the bottom 50% of the income distribution, being the welfare changes almost irrelevant for households located within percentiles 50 and 70. As expected in a reformed economy with increased tax progressivity, households in the top part of the income distribution are those who suffer the largest welfare losses. However, it is particularly curious that welfare losses affect households populating the 8th decile and especially those located in the top 10% of the income distribution, but they do not affect the group of households censored in the 9th decile. Surprisingly, not only households in this particular decile do not experience welfare losses, but some of them even find positive, albeit very small welfare variations. What forces could be driving this particular distribution of welfare changes along the income distribution of households? A narrative to rationalize these findings could be found by looking at Panels E and F, where the working and retired households are sorted by income, respectively.

It can be appreciated how the lowest decile of the income distribution is solely populated by retired households, since their minimum income (transfers, $\omega$) is always lower than the income received by the least efficient working household ($s=1$). Then, since these retired households in the lowest income decile experience welfare gains, the aggregate welfare change of households in this decile is positive. The aggregate welfare changes observed for households populating the income distribution from percentile 10 to percentile 50 are largely positive due to welfare gains of both working and non-working households. These agents directly benefit from a more progressive tax system that levies less taxes on lower-income households. Consequently, households beyond the 50th percentile of income, either working or retired, are losers in this reform of progressivity. As one might expect from such a reform, these losses are increasing in income. That is, the higher the income, the higher the tax rate faced by households. Even so, for working households there exist a couple of exceptions that are worth mentioning.

Certain peculiarities can be observed in the income distribution of working households. It can be seen how working households do not appear in the first decile. Moreover, the most productive working households, i.e., those receiving efficiency shocks $s=3$ and $s=4,$ are solely located in the top decile of the distribution. In the rest of the distribution, only the least productive households appear (those receiving shock $s=1$) and only beyond the median appear those that are somewhat more productive than the previous ones (those receiving income shock $s=2$) but not as much as the most productive ones. This is reasonable, since the most productive households account for a small fraction of the stationary distribution of working households and their endowments of efficiency labor units are between 7 and 9 times greater than those of the agents receiving the worst shock. However, there are two particularities in the income distribution of these working households that must be highlighted. First, conversely to what one could expect, agents receiving shock $s=1$ in the second decile of the income distribution present welfare losses. These households could be the typically very poor and inefficient ones, whose wages are very low and are subject to a further reduction due to the general equilibrium effects of the reform. Second, some working households in the 6th and 9th deciles of the income distribution who receive income shock $s=2$, unlike the rest of the households in the same percentiles, experience an increase in their welfare instead of a decrease, which goes against what one might expect when increasing progressivity. These particular working households suffer from the reform through the channel of higher taxes, but the higher income from capital that they receive due to a higher interest rate produces welfare gains that overpass the previously mentioned losses. The increase in the interest rate, which raises their capital returns, outweighs the negative fallout suffered by these households derived from a higher tax rate.

*Sorting by value function* It is hard to determine who benefits more from the optimal reform sorting the households according to their wealth or according to their before-tax-after-transfers income. This is because permanent income is a function of both financial wealth and human wealth. Alternatively, in Panel G households are ranked by their value function, as it reflects their expected lifetime value given their individual state (*a*, *s*). This could be the best way to rank the households since they derive utility from both wealth and income at the same time. The welfare changes are positive for every household populating the bottom 70% of the value function distribution, being these positive variations increasing up to the 30th percentile and decreasing beyond it. This narrative is justified by what is shown in Panels H and I, which report the welfare changes for working and non-working households, respectively. The increasing aggregate welfare gains observed along the first three deciles are mainly driven by the welfare changes experienced by retired households and the least productive working agents. As expected, those households are the ones who benefit more from the increase in progressivity as they will face lower personal income tax rates, irrespective of their income coming through the capital or through the labor/pension channel. Beyond the 30th and up to the 70th percentile of the value function distribution, welfare gains are decreasing in value function. The households populating this area of the distribution, in which working households receiving shock $s=2$ begin to appear, earn more income than those in the bottom 30%, thus being their welfare gains lower, but still positive, as they face higher tax rates. When it comes to analyzing households situated within percentile 70 and 90 of the value function distribution, one can see the difficulty to find a clear pattern. These households would be the ones who experience the largest trade-off between (i) positive welfare effects derived from higher income (due to an increased interest rate that pushes up capital returns) and (ii) adverse effects emerging from their relatively large income being taxed at a higher rate (due to the increase in progressivity of the income tax schedule). Finally, the most productive households (working agents receiving shocks $s=3$ and $s=4$) and the wealthiest ones (being those either working or retired) are concentrated in the top 10% of the value function distribution. As expected, these households face the largest welfare losses. It can be here noticed that the progressivity reform benefits a large part of the population while penalizing the efficiency of the economy and the income generation processes, as the most efficient households and those with the highest value function position are the ones who finance the reform and experience the largest welfare losses.

### Effects on macroeconomic and fiscal aggregates

**Fig. 4:**
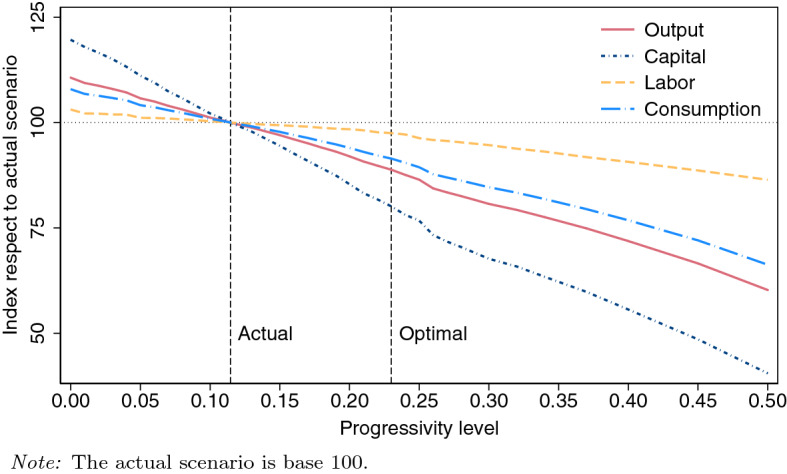
Aggregate welfare change

For each reformed economy evaluated in the progressivity grid $\tau =\{0.00,\ldots , 0.50\}$, the main macroeconomic aggregates are calculated. According to this, the evolution of these magnitudes on progressivity is depicted in Fig. 4. The aim is to observe the behavior of these aggregates with respect to the progressivity of the personal income tax. Note the fiscal ratios (i.e., *T*/*Y*, *Tr*/*Y*, and *G*/*Y*) will remain unchanged by construction of the reform.

Broadly speaking, it is clear that aggregate capital and output are decreasing in progressivity in a (almost) linear pathway, with the drop in capital being more pronounced than in output. On the other hand, aggregate consumption and aggregate labor are also decreasing in progressivity. However, they do not fall in the same proportions as capital or output do. Aggregate labor is the magnitude that decreases in the smallest proportion across economies with different levels of progressivity. These results confirm the traditional belief behind these type of progressivity reforms.

A one-to-one comparison between the main macroeconomic aggregates and ratios of the baseline economy, $E_{BE}$, and those of the optimally reformed economy, $E_{0.23}$, is reported in Table 5. This is simply a comparison between the steady states of these two economies, which differ from each other in the parameter of progressivity, $\tau$, and in average level of taxes, $\lambda$, chosen by the government in order to keep fiscal ratios constant while establishing a different progressivity level. The percentage change in these magnitudes between these two economies is reported in the third row of the table.

**Table 5:** Macroeconomic and fiscal aggregates and ratios

Economy	*Y*	*K*	$L^{\mathrm{a}}$	$H^{\mathrm{b}}/\ell$	*K*/*L*	*L*/*H*	*Y*/*H*	*K*/*Y*	*I*/*Y*	*G*/*Y*	*T*/*Y*	*Tr*/*Y*
$E_{BE}$	11.29	48.12	3.03	30.78	15.88	3.08	11.46	4.26	22.00	22.27	33.51	11.24
$E_{0.23}$	10.02	38.55	2.96	29.66	13.05	3.11	10.56	3.85	19.85	22.27	33.51	11.24
% change	$-$ 11.25	$-$ 19.89	$-$ 2.31	$-$ 3.64	17.84	1.17	$-$ 7.86	$-$ 9.79	$-$ 9.80	0.00	0.00	0.00

$^{\mathrm{a}}$ *L* denotes aggregate labor input

$^{\mathrm{b}}$ $H/\ell$ denotes the share of disposable time allocated to market activities

The optimal reform implies that the progressivity level must be increased from $\tau =0.1146$ to $\tau =0.23$, which means that, in order for the government to maintain a balanced budget policy and constant *Tr*/*Y* and *G*/*Y* levels, the average level of taxes must be increased from $\lambda =0.8924$ to $\lambda =1.18$ and the normalized transfers to retirees must drop from $\omega =3.22$ in the baseline economy to $\omega =2.86$ in the optimally reformed economy. This fact causes wages, *w*, to fall from 1.95 to 1.78 and the interest rate, *r*, to go from 0.0599 to 0.0720. As shown in Table 5, increasing progressivity to its social optimum means that aggregate labor and the share of disposable time allocated to working activities are reduced. This is a directly derived result from an increasing taxation of labor income for the rich and a decreasing taxation for the poor, which discourages aggregate labor (as the richest households are those working the most) and makes many households opt to enlarge their leisure time, which they also derive utility from. This causes productivity per worker, denoted by *Y*/*H*, to fall. Likewise, aggregate capital falls because the government, by increasing progressivity, is taking away precautionary motives for households to save for self-insurance. Thus, by lowering their savings rate, they accumulate less capital, which reduces the investment to output ratio, *I*/*Y*. Consequently, aggregate capital falls, which induces the previously discussed rise in the interest rate due to a diminished asset supply. Further, aggregate output would also suffer a setback, since it is defined in terms of aggregate capital and aggregate labor, factors that, as mentioned above, fall when progressivity increases. Therefore, it can be interpreted that an increased level of progressivity in the personal income tax scheme enlarges the distortion in the intertemporal allocation of consumption, which encourages households to work less and to save to a lower degree.

### Effects on income and wealth inequality

The Gini indexes and the Lorenz curves of income and wealth in the baseline economy, $E_{BE}$, and in the optimally reformed economy, $E_{0.23}$, are reported in Table 6. As shown by the Gini indexes, the increase in progressivity from $\tau =0.1146$ to $\tau =0.23$ entails a drop in both wealth and income (before all taxes and after transfers) inequality, being the latter decrease less accentuated.

The effect of the reform on the income distribution is not difficult to interpret. As income tax progressivity increases, households with a higher share of total income are those that suffer from the reform and those that experience a fall in their share, i.e., households beyond the 80th percentile lose about 4% of their share on average. On the other hand, households populating the bottom 60% of the distribution (the income-poor and medium-income) witness how their share of total income increases by 6–8%. Finally, households situated in the 4th quintile of the income distribution do not experience any variation in their income share. These results can by rationalized by a twofold effect: (i) lower aggregate income in the reformed scenario driving the share of the poorest households up and (ii) higher taxation of the richest households in favor of lower taxation of the poorest households in income. These findings show a considerable redistributive power of the reform in terms of income distributional measures, with an income Gini coefficient decreasing from 0.45 to 0.42.

**Table 6:** Distributions of income and wealth

Economy	Gini	Percentiles (%)				Top groups (%)		
		$<40$	40–60	60–80	80–100	90–95	95–99	99–100
*The distribution of income (before all taxes and after transfers)*								
$E_{BE}$	0.45	14.72	13.72	21.32	50.24	10.85	13.35	13.57
$E_{0.23}$	0.42	15.96	14.55	21.40	48.09	9.95	12.81	12.73
% change	$-$ 5.60	8.41	6.09	0.36	$-$ 4.28	$-$ 8.32	$-$ 4.04	$-$ 6.15
*The distribution of wealth*								
$E_{BE}$	0.68	3.80	9.32	17.45	69.43	13.54	19.68	19.63
$E_{0.23}$	0.56	7.57	13.41	21.47	57.55	12.10	15.10	14.21
% change	$-$ 16.62	99.05	43.98	23.02	$-$ 17.11	$-$ 10.66	$-$ 23.28	$-$ 27.62

This redistributive power of the reform is even stronger when looking at the wealth distribution. The least wealthy households are those that gain share of the total wealth to a wider extent. Households below the 40th percentile of wealth increase by almost 100% their share, while households situated in percentile groups 40–60 and 60–80 experience a 44% and a 20% increase, in their share of total wealth after the reform, respectively. On the other hand, the wealthiest households, and especially those located in the top 5% of the distribution, experience very large decreases in their wealth share. These consequences may be such because the reform discourages savings of the wealth-rich more than it does with the wealth-poor savings. Wealthy people will be taxed more on their increased incomes (due to a higher interest rate) and will have more incentive than poorer people to lower their savings rates. This causes the distribution of wealth to be more egalitarian, with the wealth-poor having more share and the wealth-rich having a lower share.

**Fig. 5:**
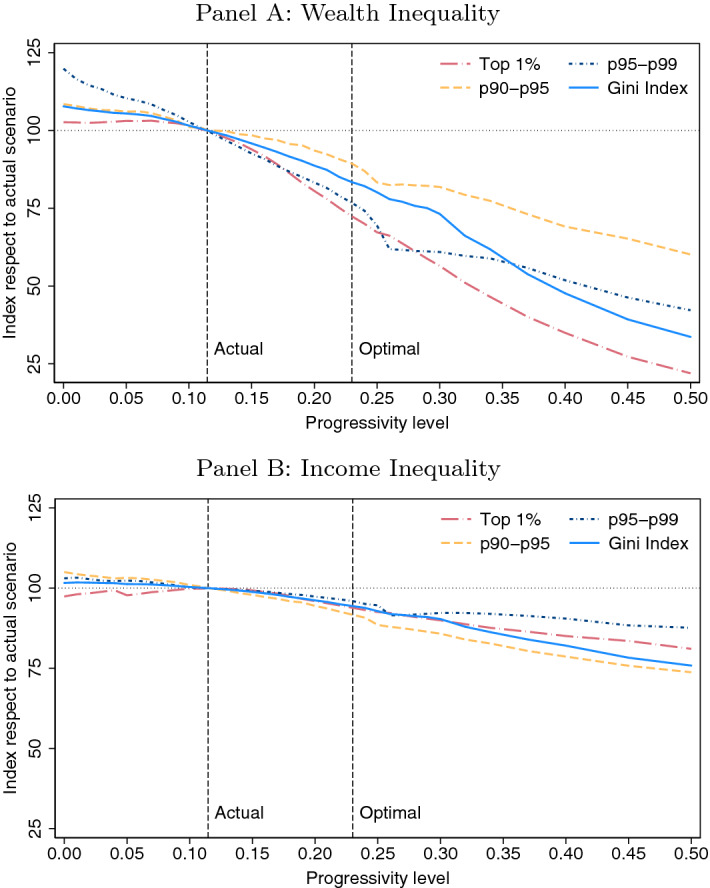
Inequality levels

Further, in order to deepen the understanding of the relationship between income and wealth inequality and progressivity, it may be convenient to review Fig. 5, where the levels of inequality are reported for each reformed economy evaluated in the progressivity grid $\tau =\{0.00,\ldots , 0.50\}$.

As expected in implementing this type of reforms, the Gini indexes and the income- and wealth-top shares are continuously decreasing in progressivity, being this decrease more pronounced in the income case. In Panel A, looking at the relationship between measures of wealth inequality and income tax progressivity, one can see how the share of the top 1% households is the one that decreases the most in progressivity. Further, the share of the households censored at the wealth percentile group 95–99 decreases to a lesser extent than the top 1% one, and accordingly, the share of the households censored at the percentile group 90–95 decreases to a lesser extent than the share of the percentile group 95–99. These share variations coincide with what one could expect in advance. However, when looking at the relationship between measures of income inequality and income tax progressivity in Panel B, it can be seen how the pattern observed in the wealth-top shares is not followed in the case of income-top households. Surprisingly, the top 1% households of the income distribution experience an income share decrease in progressivity which is less accentuated than that faced by households populating the income distribution between the 90th and the 95th percentiles.

### Who pays the reform? Effects on the personal income tax scheme

Once the optimal progressivity reform has been analyzed in terms of welfare, macroeconomic aggregates, and inequality, a relevant issue to approach is how this optimal level of progressivity would affect the Spanish taxpayers. For that purpose, making use of the aforementioned Spanish tax microdata, the potential impact of setting the optimal progressivity level on the Spanish taxpayers is analyzed.

**Table 7:** Percent change in the effective average tax rate

	Income percentiles (%)					Income top groups (%)		
	$<20$	20–40	40–60	60–80	80–100	90–95	95–99	99–100
% Change	$-$ 53.42	$-$ 34.64	$-$ 16.17	$-$ 5.33	4.04	3.68	12.13	16.21

Using the output of the model economy here presented, it is possible to compute what is the change in the effective average tax rate derived from implementing the optimal progressivity reform in the personal income tax scheme for each household percentile group of the income distribution. In summary, the optimal percent change in the effective average tax rate faced by each group of households of the income distribution in the model economy is reported in Table 7.

In a similar way, the actual effective average personal income tax rate can be computed for the Spanish economy using household level tax microdata. Then, applying the optimal percent changes in the effective tax rate computed for 100 points of the income distribution in the model economy to the same 100 points of the actual Spanish income distribution obtained with tax microdata, it can be calculated how much personal income taxes the Spanish households would have to pay if this reform were implemented in reality.

**Fig. 6:**
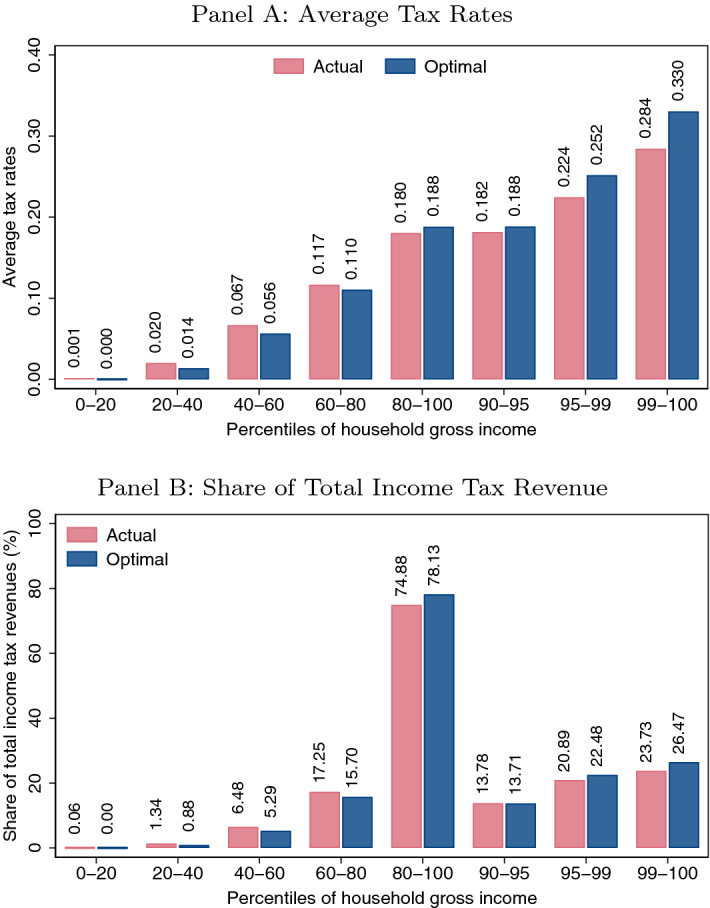
Effective average tax rates and share of tax revenues

In the Panel A of Fig. 6, these effective average tax rates are reported by percentile groups of household gross income for the actual Spanish economy. Then, these numbers are compared with the average tax rates that the same households would face in the optimal scenario.^21^ Households in a region lower than the 20th percentile would continue to face very small or no average tax rates. However, households between the 20th and the 80th percentiles would experience a decrease in their average tax rates. More precisely, the effective average tax rate encountered by a household situated within the 40th and the 60th percentiles would drop from 0.067 to 0.056, which involves a change of 1.1 p.p. On the other hand, households above the 80th percentile would experience a drastic increment in their effective average tax rate. For instance, the top 1% households of the gross income distribution would go from confronting an average tax rate of 0.284 in the actual scenario to dealing with an average tax rate of 0.330 in the optimal one. These changes would imply that the tax returns of households located in the top 20% of the income distribution would account for the 78.13% of the total income tax revenue instead of the actual 74.88%, as shown in Panel B. Particularly interesting is that the tax payments of the top 1% households of the gross income distribution would account for 26.47% of the total personal income tax revenue under the optimal reform, an increase of 2.74 percentage points with respect to the actual scenario. These results reveal that the optimal reform would be financed by the income-rich working households.

## Concluding remarks

In this paper, a heterogeneous households general equilibrium model featuring both life cycle and dynastic elements is calibrated to replicate some relevant characteristics of the Spanish economy and used to evaluate potential reforms of the income tax system. Each of these reforms involves setting a different level of progressivity, $\tau$, in the personal income tax. Further, in each reformed economy the government must choose a new average level of taxes, $\lambda$, such that, for each different progressivity level, $\tau$, it balances its budget and guarantees the same level of public expenditure, *G*/*Y*, and transfers, *Tr*/*Y*.

The results of these evaluations reveal that elevating progressivity to a higher level than the current one could generate aggregate welfare gains. More precisely, the optimal reform of the progressivity level would be the one which maximizes the aggregate welfare from the point of view of a Benthamite social planner. This planner takes into account all people in the economy in the same way. As a result of this welfare maximization setup, the optimal reform of the personal income tax would involve raising progressivity from 0.1146 (its current value) to 0.23. Such reform would induce that, on average, every households in the economy would increase its consumption by 3.08%.

By decomposing the aggregate welfare change, it is shown that most of the welfare gains are obtained by direct improvements in the tax system, which means that most of the aggregate welfare gains come from poorest households facing lower effective income tax rates and richest households affronting higher effective income tax rates. Contrarily, the general equilibrium effects of the optimal reformed economy (higher interest rate and lower wage) and the effects resulting from changes in the equilibrium distribution of households across income levels (larger mass of households at lower income levels) show a welfare loss, but these losses are so small that together cannot overpass the welfare gains directly coming from the reformed tax system, jointly resulting in positive aggregate welfare changes. In a next step, these welfare gains are decomposed by household type, where it is observed that the poorest working and non-working households are the ones who benefit the most from the reform. Contrarily, the most efficient working households and the wealthiest ones (either working or non-working) are those who experience the largest trade-off between (i) positive welfare effects derived from higher income (due to an increased interest rate that pushes up capital returns) and (ii) adverse effects emerging from higher tax payments (due to the increase in progressivity of the income tax that discourages labor and savings). The losses from this trade-off are particularly high in top parts of the income and wealth distributions and clearly offset the potential welfare gains of the households populating such areas. Therefore, knowing that these agents would be the losers of the reform, despite positive aggregate welfare effects, the consequences on aggregate capital, labor, and output would be negative, which means that the economy would experience an efficiency loss. Lastly, looking at the distributional implications, this reform would reduce income and wealth inequality.

Finally, the theoretical results are evaluated with Spanish tax microdata. From the point of view of a Benthamite social planner, households between the 20th and the 80th percentiles would experience a decrease in their average tax rates under the optimal progressivity reform. For example, the effective average tax rate encountered by a household situated within the 40th and the 60th percentiles of the income distribution would drop from 0.067 to 0.056, which involves a change of 1.1 p.p.. On the other hand, households above the 80th percentile would experience a drastic increment in their effective average tax rate. For instance, the top 1% households of the household gross income distribution would go from confronting an average tax rate of 0.284 in the actual scenario to dealing with an average tax rate of 0.330 in the optimal one.

In conclusion, as policy implications arising from this study, what the model (jointly with the data) indicates is that, in terms of aggregate welfare, it would be optimal to increase the progressivity of the personal income tax. In addition, the reform would reduce income and wealth inequality. However, this would lead to a efficiency loss of the economy, since it discourages work and savings mainly by penalizing the top-working and wealthiest households.

One should keep in mind that the analysis presented here is focused on the taxation of personal income. And within this scope, the study is focused on welfare gains derived from changes in personal income tax progressivity. More precisely, the study investigates the progressivity of the income tax which is welfare-maximizing. A different question is what tax progressivity would lead to the maximization of income tax revenues, i.e., calculating the income tax Laffer curve. In this sense, Guner et al. (2020) estimate, at the individual level (not at the household level, as it is done in this work) that the revenue-maximizing income tax progressivity would be $\tau =0.19$. Bearing in mind that income tax progressivity is somewhat higher for individuals than for households (due to the compensation of higher incomes of the principal earner with lower incomes of the rest of the earners), one could think that revenue-maximizing income progressivity at the household level could fall within the vicinity of $\tau =0.15$. Finally, it should be noted that the analysis presented here does not consider transitional dynamics, but is merely a comparison between steady-states of the current scenario and the optimal one. Therefore, it should be taken into account that a change in progressivity would take several periods for the capital and other macrovariables to adjust to the levels of the optimal steady-state.

For future research, there are several lines of investigation open. One of them would be to adapt the model so that it incorporates a specification for the budget constraint that parametrizes each tax separately (consumption tax, estate tax, social contributions, etc.), as it is done by Guner et al. (2020). Another line would be to make the transfers or the retirement benefit progressive. This could improve the theoretical setup and make it suitable for evaluating other policies such as the universal basic income or the negative income tax. Beyond this, there is potential work to be done in finding transition paths between steady states (for example, introduction of endogenous retirement decisions), which could vary the optimal progressivity found by the model, as argued by Bakis et al. (2015). It would be interesting to adjust the model to cover other types of methodological literature as well, which could make the results of the model more robust. For example, extend it to incorporate aggregate shocks to the economy (business cycles), to have heterogeneity in firms (some degree monopolistic power) or to introduce heterogeneity in the marginal propensities to consume of individuals, which could be related to define different preferences for different households.

## Transition between states

### Transition between retirees and descendants

This subsection explains how $\Gamma _{{\mathcal {R}}{\mathcal {E}}}$ is computed and how $\phi _1$ and $\phi _2$ affect such transition submatrix. Let $p_{ij}$ denote the transition probability from $i \in {\mathcal {R}}$ to $j \in {\mathcal {E}}$, let $\gamma ^{*}_{i}$ be the invariant measure of households that receive shock $i \in {\mathcal {E}}$, and let $\phi _1$ and $\phi _2$ be the two parameters that shift the probability mass toward the diagonal and toward the first column of the submatrix $\Gamma _{{\mathcal {R}}{\mathcal {E}}}$, then the recursive procedure that is used to compute the $p_{ij}$ is the following:

*Step 1* First, $\phi _1$ is used to shift the probability mass from a matrix with vector $\gamma ^{*}_{{\mathcal {E}}}=(\gamma ^{*}_{1},\gamma ^{*}_{2},\gamma ^{*}_{3},\gamma ^{*}_{4})$ in every row toward its diagonal, as follows:

$$\begin{aligned} p_{51}= & {} \gamma ^{*}_{1}+\phi _1\gamma ^{*}_{2}+\phi ^{2}_{1}\gamma ^{*}_{3}+\phi ^{3}_{1}\gamma ^{*}_{4} \\ p_{52}= & {} (1-\phi _1)[\gamma ^{*}_{2}+\phi _1\gamma ^{*}_{3}+\phi ^{2}_{1}\gamma ^{*}_{4}] \\ p_{53}= & {} (1-\phi _1)[\gamma ^{*}_{3}+\phi _1\gamma ^{*}_{4}] \\ p_{54}= & {} (1-\phi _1)\gamma ^{*}_{4} \\ p_{61}= & {} (1-\phi _1)\gamma ^{*}_{1} \\ p_{62}= & {} \phi _1\gamma ^{*}_{1}+\gamma ^{*}_{2}+\phi _{1}\gamma ^{*}_{3}+\phi ^{2}_{1}\gamma ^{*}_{4} \\ p_{63}= & {} (1-\phi _1)[\gamma ^{*}_{3}+\phi _1\gamma ^{*}_{4}] \\ p_{64}= & {} (1-\phi _1)\gamma ^{*}_{4} \\ p_{71}= & {} (1-\phi _1)\gamma ^{*}_{1} \\ p_{72}= & {} (1-\phi _1)[\phi _1\gamma ^{*}_{1}+\gamma ^{*}_{2}] \\ p_{73}= & {} \phi ^{2}_1\gamma ^{*}_{1}+\phi _{1}\gamma ^{*}_{2}+\gamma ^{*}_{3}+\phi _{1}\gamma ^{*}_{4} \\ p_{74}= & {} (1-\phi _1)\gamma ^{*}_{4} \\ p_{81}= & {} (1-\phi _1)\gamma ^{*}_{1} \\ p_{82}= & {} (1-\phi _1)[\phi _1\gamma ^{*}_{1}+\gamma ^{*}_{2}] \\ p_{83}= & {} (1-\phi _1)[\phi ^{2}_1\gamma ^{*}_{1}+\phi _1\gamma ^{*}_{2}+\gamma ^{*}_{3}] \\ p_{84}= & {} \phi ^{3}_1\gamma ^{*}_{1}+\phi ^{2}_{2}\gamma ^{*}_{2}+\phi _{1}\gamma ^{*}_{3}+\gamma ^{*}_{4} \end{aligned}$$

*Step 2* Then for $i=5,6,7,8$, parameter $\phi _2$ is used to shift the resulting probability mass toward the first column as follows:

$$\begin{aligned} p_{i1}= & {} p_{i1}+\phi _2 p_{i2}+\phi ^{2}_{2} p_{i3}+\phi ^{3}_{2} pi_{i4} \\ p_{i2}= & {} (1-\phi _2)[p_{i2}+\phi _2 p_{i3}+\phi ^{2}_{2} p_{i4}] \\ p_{i3}= & {} (1-\phi _2)[p_{i3}+\phi _2 p_{i4}] \\ p_{i4}= & {} (1-\phi _2)p_{i4} \end{aligned}$$

### Joint age and endowment stochastic process

The transition matrix between all possible states of the model economy is presented in Table 8. This is the Markov Chain transition matrix featuring the stochastic process that jointly defines the age and the endowment of labor efficiency units of each household and the complete invariant distribution of households, $\gamma ^{*}$. A more detailed definition of each of the submatrices found in this matrix can be reviewed in Sect. 2.1.

**Table 8:** Stochastic process for the endowment of efficiency labor units and age

	*e*(*s*)	$\gamma ^{*}$	$\Gamma _{{\mathcal {S}}{\mathcal {S}}}$ from *s* to $s'$							
			$s'=1$	$s'=2$	$s'=3$	$s'=4$	$s'=5$	$s'=6$	$s'=7$	$s'=8$
$s=1$	1.00	19.43	87.02	10.06	0.01	0.05	2.86	0.00	0.00	0.00
$s=2$	2.71	35.18	2.35	93.78	1.00	0.01	0.00	2.86	0.00	0.00
$s=3$	7.80	5.59	0.01	3.50	93.59	0.04	0.00	0.00	2.86	0.00
$s=4$	90.00	0.35	0.01	1.68	0.01	95.44	0.00	0.00	0.00	2.86
$s=5$	0.00	12.66	4.39	0.01	0.02	0.01	95.61	0.00	0.00	0.00
$s=6$	0.00	22.92	4.26	0.13	0.02	0.02	0.00	95.61	0.00	0.00
$s=7$	0.00	3.64	4.14	0.12	0.13	0.01	0.00	0.00	95.61	0.00
$s=8$	0.00	0.23	4.03	0.12	0.23	0.13	0.00	0.00	0.00	95.61

*e*(*s*) denotes the relative endowments of efficiency labor units; $\gamma ^{*}$ denotes the stationary distribution households; $\Gamma _{{\mathcal {S}}{\mathcal {S}}}$ denotes the transition probabilities of the process on the endowment of efficiency labor units and age

## Full list of parameters

The setup of this model economy has 36 parameters. A full description of the parameters and how they are calibrated or estimated is contained in the body of the paper. For convenience, a complete list of parameters is provided here. It is also indicated which ones are normalizations, calibrated, or estimated.

**A full list of the parameters:**

- 5 parameters to describe the household’s preferences
  $$\beta $$, $$\sigma $$, $$\varphi $$, $$\chi $$, $$\ell $$
- 2 parameters for production technology
  $$\alpha $$, $$\delta $$
- 4 parameters for the government policy
  $$\omega $$, $$\lambda $$, $$\tau $$, $$\kappa $$
- 25 parameters for the joint process on age and endowment of efficiency labor units
  *J*, $$p_r$$, $$p_s$$, $$\phi _1$$, $$\phi _2$$
- 5 so far, and 20 more for the submatrix $\Gamma _{{\mathcal {E}}{\mathcal {E}}}$ and the endowments of efficiency labor units
  $$e(s)=\left[ e(1),e(2),e(3),e(4),0,0,0,0\right] $$
  (4 here: *e*(1), *e*(2), *e*(3), *e*(4))
  $$\Gamma _{{\mathcal {E}}{\mathcal {E}}}=\left[ \gamma _{11},\gamma _{12},\gamma _{13},\gamma _{14};\gamma _{21},\gamma _{22},\gamma _{23},\gamma _{24};\gamma _{31},\gamma _{32},\gamma _{33},\gamma _{34};\gamma _{41},\gamma _{42},\gamma _{43},\gamma _{44}\right] $$ (and 16 here)

**Normalization, 7 parameters:** $J=4$, $\ell =3.2$, $e(1)=1$ (endowment for the least productive households), and four normalizations on the rows of $\Gamma _{{\mathcal {E}}{\mathcal {E}}}$ (so that each rows adds up to one).

**Directly identified, 8 parameters:** $\sigma$, $\varphi$, $\lambda$, $\tau$, $p_r$, $p_s$, $\alpha$, $\delta$

**Estimated by simulated method of moments, 21 parameters:**

Those for preferences, government, and taxation: $\beta$, $\chi$, $\omega$, $\kappa$

And 17 parameters for the joint process on age and endowments of efficiency labor units:

$\phi _1$, $\phi _2$, $e(s)=\left[ e(1),e(2),e(3),e(4),0,0,0,0\right]$ (3 here, since $e(1)=1$ is a normalization of the least productive household)

and $$\Gamma _{{\mathcal {E}}{\mathcal {E}}}=\left[ \gamma _{11},\gamma _{12},\gamma _{13},\gamma _{14};\gamma _{21},\gamma _{22},\gamma _{23},\gamma _{24};\gamma _{31},\gamma _{32},\gamma _{33},\gamma _{34};\gamma _{41},\gamma _{42},\gamma _{43},\gamma _{44}\right] $$

(12 here, which are the off-diagonal elements, as the four normalizations must be done for the diagonal elements)

Note that the choice to use the diagonal elements of $\Gamma _{{\mathcal {E}}{\mathcal {E}}}$ as the elements to be normalized has an important advantage in computation. Since the off-diagonal elements are smaller, by having the diagonal elements given by whatever was leftover to make the row sum up to one, the problem that they may end up being negative is avoided.

## Computation

This section describes the computation of the model. First, it describes how the value functions and the stationary distribution are computed. Second, it provides a brief description of how the model moments are calculated. Third, it sheds some light on the calibration of such model economy. Finally, it clarifies the process to get the general equilibrium of the model. It is particularly important to mention that the codes are written and executed in MATLAB in the spirit of the Value Function Iteration algorithm developed by Kirkby (2017).

All simulation exercises involved a burn-in of 1000 points (typically starting from the “mid-point” of the relevant distribution).

### Value functions and stationary distribution

To calculate the optimal decision rules, the state space is discretized and a value function iteration using the Howard’s improvement algorithm is performed.

The size of the state space is $n_a\times n_s =606 \times 8 =4,848$ points. The size of the control space is $n_a \times n_h = 606 \times 13=7,878$ points. Since the numbers of working-age and retirement states are $n_{{\mathcal {E}}} = n_{{\mathcal {R}}} = 4$, the total number of search points is $\left[ (n_a \times (n_{{\mathcal {E}}} + n_{{\mathcal {R}}}))\times (n_a \times n_h)\right] =38,192,544$.

The stationary distribution is approximated with a discretization of the associated distribution function. The grid for this approximation is the same as that used to solve for the value function. The stationary distribution is calculated by iterating on the whole distribution, using the optimal policy functions and the transition matrix of the idiosyncratic shocks (exogenous process that determines the age and the endowment of efficiency labor units of the households). This is done until it converges, as measured by a distance criterion based directly on the monotone mixing condition underlying the theory that ensures that a stationary distribution exists.^22^ This process is more demanding computationally than those typically used for calculating the stationary distribution, but that is important since the model moments relating to the top percentiles, e.g., the asset share of the top decile of asset holders, otherwise varied substantially between different simulations (much less simulations were fine for giving stable results for the first moments, such as the capital stocks, but the top percentile moments can be quite volatile).

### Model moments

The distributional and aggregate moments or statistics of the model economy can almost all be computed directly as integrals with respect to the stationary distribution of households. It means that this just involves taking weighted sums, as the stationary distribution is approximated as a weight for each point on a grid. The only exceptions are those moments that measure the earnings life cycle profile and the intergenerational correlation of earnings. These particular computations are presented in what follows straight after.

*Life cycle profile of income* This is measured as the ratio of the average income of agents between ages 45 and 49 (senior workers) to that of agents between ages 25 and 29 (new entrants). To compute this particular measure, a random newborn is drawn from the distribution of newborns in a first step.^23^ Then, the household is simulated for 30 periods recording its productivity in each year and recording both its average earnings between ages 25 and 29 and those between ages 45 and 49. Note that households in the model are born when they enter the labor market, at age 20. Therefore, following the household for 30 periods means following it up to age 50. It is done for a large number of households and, after dropping those ones retired before reaching the old phase, the average ratio across remaining households is calculated.^24^

*Intergenerational correlation of earnings* This is measured as the correlation between the average annual earnings of two consecutive generations of the same dynasty. In order to compute this moment, a random newborn is drawn from the distribution of newborns in a first step (see footnote 23). Next, the household is simulated recording its annual earnings until it dies twice. Note that households are followed during 40 periods, i.e., from birth at age 20 up to age 60. Then, on the basis of this, the average annual earnings for the first and second generations of the dynasty are calculated. This process is done for a large number of households and then the correlation between average annual earnings of both generations is calculated.^25^

### Calibration

As previously commented in the document, the setup of this model economy has 36 parameters. Seven of them are normalizations and other 8 are directly identified. This leaves 21 parameters, which are estimated using the simulated method of moments. It means that the values of these 21 parameters are those that minimize the distance between 22 moments of the model and the same 22 moments for the Spanish economy.^26^

This model is a general equilibrium setup. Note that since aggregate production is depicted by a Cobb–Douglas production function, and due to the assumption of perfect competition implying that the interest rate equals the marginal product of capital, it is possible to identify the interest rate in terms of *K*/*Y*, $\alpha$, and $\delta$.^27^ Thus, given that one target is *K*/*Y*, and since $\alpha$ and $\delta$ are directly identified, the value that the interest rate must take in equilibrium can be calculated. This feature is exploited in the calibration process. By taking the interest rate as an input, the target value on *K*/*Y* becomes in effect the general equilibrium condition. In other words, if a large weight is placed on the *K*/*Y* moment, the general equilibrium condition is being stressed throughout the calibration process. This little trick avoids the need to loop over the calculation of general equilibrium conditions all along. After reaching a certain arbitrary level of convergence the calibration process can be considered as completed. Thereafter, the general equilibrium must be calculated, as explained in Sect. C.4, with the calibrated parameters resulting from this process.

In the following, the implementation of the simulated method of moments to estimate the 21 aforementioned parameters is detailed.

*Step 1* A vector of 22 weights is chosen, one for each of the 22 moments. These weights measure the relative importance of each of the specified moments. In this sense, a greater weight is placed on the capital-to-output ratio, since, as commented before, this represents the condition of general equilibrium. In turn, the life cycle profile of earnings and the intergenerational correlation of earnings carry less weight, since neither of them is a clear map from the model to the data. (The first due to stochastic aging and the second because what is really measured in the data is the correlation between parents and children.) A greater weight is placed on the ratio of transfers to output, as this calibrates the parameter $\omega$, namely the endowment of retirees, which plays a major role in the analysis. On the other hand, we should not put too much weight on *G*/*Y*, since it is defined as a leftover difference between *Tr*/*Y* and *T*/*Y* and is therefore closely related to other targets. Finally, the weights that the measures of inequality (distributional statistics) have are raised but not too much, since these parameters account for more than half of the total number of parameters.

*Step 2* A guess is made for the values of the 21 parameter unknowns. At the time of implementing the guess, the initial values are based on the existing literature at first, and then, they are based on previous runs of the code that were getting closer and closer to the level of convergence. This step took the most time.

*Step 3* The optimal policy function and the stationary household distribution are computed (given the interest rate and parameter values).

*Step 4* The 22 moments of the model economy are computed (given the interest rate and parameter values).

*Step 5* It checks whether the weighted distance of the model moments from the data moments is small enough. If the level of arbitrary convergence is reached, the calibration is completed. If not, new values are chosen for the parameters and return to step 3. (For this computational task the CMA-ES algorithm described below is used.)

*Step 6* Once these parameters have been estimated, the general equilibrium of the model is calculated. (See Sect. C.4.)

The loop used to calibrate the parameters by matching the model moments to the data moments is implemented using the Covariance-Matrix Adaptation - Evolutionary Strategy (CMA-ES) algorithm. For a more involved explanation see Andreasen (2010), who also provides a MATLAB code with the implementation of the algorithm. This algorithm outperforms many other inbuilt MATLAB optimization functions (fgoalattain, fminsearch, fminunc, fmincon, among others) in the particular task of calibrating this model. This algorithm works by starting out considering the entire parameter space. Parameter vectors are drawn randomly (based on the covariance-matrix and a focal-point) and evaluated. Based on these evaluations, the covariance-matrix and focal-point are updated. As the algorithm progresses the average distance between the parameter vectors drawn and the focal-point is progressively reduced. Once certain convergence criterion is met, the focal-point is returned as the estimated value of the true parameter vector. For running this subroutine, the lower and upper bounds of the parameter space and the size of the step in the parameter search must be specified.

### General equilibrium

The calculation of a general equilibrium in this family of models typically involves finding an interest rate which induces individual behavior which generates aggregate variables (like output, labor, or capital) that in turn lead back to the original interest rate. A prominent example of this is Aiyagari (1994). In this model economy, in addition to the market clearance condition (given by the interest rate), it is also required that the government balances its budget to determine the general equilibrium. These conditions result in two requirements (the so called general equilibrium equations): (i) interest rate equals the marginal product of capital ($r=\alpha K^{\alpha -1}L^{1-\alpha }-\delta$) and (ii) government balances its budget ($G/Y + Tr/Y = T/Y$).

In order to find the solution to these two general equilibrium equations, two price solvers are needed. These prices are the interest rate, *r*, and the linear term on remaining taxes, $\kappa$.^28^ In this sense, we use the general equilibrium finding stage as the calibration procedure for this parameter $\kappa$, which is previously determined by the calibration algorithm, but here it is where it takes its final calibrated value so that the government in the model economy runs a budget-balance fiscal policy. Further, in comparison with the traditional methods, there is a noteworthy difference in the way that this algorithm finds the interest rate that ensures market clearance. Here, rather than using a search algorithm on *K*/*Y* to find the general equilibrium interest rate (the standard approach), the algorithm discretizes the state space for *r* and use this to find the equilibrium value. The same procedure is applied on $\kappa$.

In summary, this algorithm calls the MATLAB fminsearch subroutine, which finds the values of *r* and $\kappa$ that respectively minimize the previously described equations. Once a certain level of arbitrary convergence has been reached, it can be said that the algorithm has found the general equilibrium.^29^

#### General equilibrium in a reformed economy

For each reform, i.e., in this study, for each level of $\tau$, a new general equilibrium needs to be calculated. To do this, the same method that was presented in the lines above is used. The only slight change is that the parameter $\kappa$ is no longer a price solver of the second general equilibrium equation anymore. Now, as each reform requires the government to keep its budget balanced and to guarantee the same level of government expenditure, *G*/*Y*, and transfers, *Tr*/*Y*, there will be three equilibrium equations. One regarding the interest rate, *r*, that will continue to be a price solver, and two more regarding the government constraints and conditions. It means that, apart from *r*, two new parameters will serve as price solvers of the general equilibrium equations, $\lambda$ and $\omega$. Beyond this, the computational procedure for the search of a general equilibrium for each new scenario is the same as the one presented above.

## Footnotes

1 The OECD average of the share of personal income tax revenues over total tax revenues (excluding social security contributions) has been around 30–35% in recent years.

2 For example, Alonso-Borrego et al. (2005) build a general equilibrium labor-search model with heterogeneous agents and firing costs for analyzing labor market reforms. Another case studies are Conde-Ruiz and González (2012) and Conde-Ruiz and González (2016), where they analyze Spain’s 2011 pension reform and evaluate potential pension system scenarios á la Bismark or Beveridge for Spain. They use a model of heterogeneous agents with overlapping generations (OLG). Again, on the subject of demographic change and pension reform in Spain, two studies are found: Sánchez Martín (2010), which uses a general equilibrium model with heterogeneous agents and endogenous retirement, and the article by Sánchez Martín and Sánchez Marcos (2010), which uses a Two-Earners OLG general equilibrium model.

3 This frames the model here presented within the methodological literature related to heterogeneous agents general equilibrium models with incomplete markets originally developed by Huggett (1993), Aiyagari (1994), Krusell and Smith (1998), Quadrini (2000), and De Nardi (2004), among others. These models (i) are devoted to account for income and wealth inequality and (ii) study decisions of households that face labor income processes that are random, household-specific, and uninsurable. In these model-based economies, households accumulate wealth in part to smooth their consumption.

4 This is crucial for quantitative evaluations of tax reforms because the tax burdens and the incentives to work and save that a particular tax scheme creates are very different at different points of the income and wealth distributions, and they affect the most on the very income-rich and wealthy households. These distributional issues are relevant in measuring the trade-offs involved in choosing between efficiency and equality of tax reforms.

5 The capital depreciation rate, $\delta$, comes from the extensively used capital law of motion, i.e., $K_t=(1-\delta )K_{t-1}+I_t$, where $I_t$ is the investment (namely, the fraction of income which is saved).

6 According to Conde-Ruiz and González (2012, 2016), the pension system in Spain seems to be very redistributive. See their estimate of parameter $\alpha$ that measures the redistributiveness of the pension system.

7 This assumption is made to reduce the computational complexity of the problem. Households receiving transfers according to their past contributions would require the inclusion of a second asset-type state variable in the household’s decision problem. In addition, in a model with endogenous labor supply, linking pensions to contributions makes the optimality condition for leisure an intertemporal decision, which increases extremely the computational costs. See Díaz-Giménez and Pijoan-Mas (2019) for a more detailed explanation.

8 The existence of an upper bound for the asset holdings is guaranteed as long as the after-tax rate of return to savings is smaller than the households’ common rate of time preference. This condition is always satisfied in equilibrium. See Huggett (1993), Aiyagari (1994), Ríos-Rull (1996), and Marcet et al. (2007) for details and proofs of this proposition.

9 Since the structure of the households’ problem is recursive, henceforth the time subscripts are dropped from all the current-period variables, and primes are used to denote the value of variables one period ahead.

10 Note that this model economy abstracts from estate taxes, i.e., there is no inheritance tax.

11 The rationale for this choice is that this value makes the aggregate labor input approximately equal to one.

12 The average household net wealth, 247,523€, is calculated using linear interpolation techniques. The Survey of Household Finances conducted by the Bank of Spain (2017, 2019) provides the average household net wealth for two particular waves, 2014 and 2017, at current prices. Using annual price changes from the World Bank (2020a), these data can be expressed in €of 2015. Then, using linear interpolation between years 2014 and 2017, the magnitude for 2015 can be computed. On the other hand, the gross domestic product per household, 58,230€, is obtained by dividing the GDP extracted from Eurostat (2020b) by the number of households. Such number of households is calculated by dividing the Spanish population quoted for the calibration reference year (46,449,565) by the average household size in Spain in the calibration reference year (2.51), both data according to Eurostat (2020c) and the Spanish National Institute of Statistics (2016), respectively.

13 Since the National Accounts do not differentiate the amount associated with private consumption expenditures in durables and in non-durables, it is assumed that the durables share in the total reported private consumption expenditures is 5%.

14 Note that the CRRA preferences would collapse to a logarithmic functional form in the case that $\sigma =1$.

15 The *Pensions at Glance* reports developed by the OECD (2015, 2017) provide data about the retirement effective age and the life expectancy at effective retirement by sex in 2014 and 2016. Then, the magnitudes for 2015 are obtained by using linear interpolation techniques. The expected duration of retirement is therefore obtained by calculating the difference between the life expectancy at effective retirement and the retirement effective age. Finally, using Eurostat (2020c) data on population by sex at effective retirement age in 2015 to ponder women and men magnitudes, the value of 22.8 years can be obtained.

16 In a previous attempt to measure such magnitude with past data, Cervini Plá (2015) reported a value for this statistic of 0.42.

17 See García-Miralles et al. (2019) for a detailed description of the estimation process and the data used. They also estimate other specifications like the one established by Gouveia and Strauss (1994). Further, they propose an alternative method to estimate these specifications by differentiating between general income (labor and some capital gains, in the Spanish legislation) and capital income.

18 The data are not censored either at the top or at the bottom of the income distribution. Recall that in Spain, the income tax return can be filled in a single or in a joint (with the spouse) way, and therefore, it is necessary to do aggregation in some cases to have the data at the household level.

19 The grid is not evenly split, but is a sparse grid with higher concentration on its central values, where the baseline and the optimal economy are encountered.

20 In this setup, in order to keep the transfers-to-output ratio constant as in the baseline economy, $Tr/Y=11.23\%$, the calibrated value for the normalized transfers to retirees, $\omega$, would drop from 3.22 in the baseline economy to 2.86 in the optimally reformed economy.

21 Note that this calculation abstracts from general equilibrium and distributional effects.
